# Targeted deletions of large syntenic regions in *Arabidopsis thaliana*

**DOI:** 10.1073/pnas.2419744122

**Published:** 2025-08-11

**Authors:** Ashot Papikian, Rachel J. Rattner, Jenni Kao, Neil Hauser, Nicholas Allsing, Allen Mamerto, Nolan T. Hartwick, Kelly Colt, Todd P. Michael

**Affiliations:** ^a^Plant Molecular and Cellular Biology Laboratory, The Salk Institute for Biological Studies, La Jolla, CA 92037; ^b^Department of Cell and Developmental Biology, School of Biological Sciences, University of California San Diego, La Jolla, CA 92093; ^c^Center for Marine Biotechnology and Biomedicine, University of California San Diego, La Jolla, CA 92037; ^d^Department of Science and Conservation, San Diego Botanical Garden, Encinitas, CA 92024

**Keywords:** CRISPR–Cas9, deletions, synteny, whole-genome duplication (WGD), dosage compensation

## Abstract

Plant genomes have undergone multiple rounds of whole-genome duplication (WGD) throughout their evolutionary history. As a result, many species, including *Arabidopsis thaliana*, retain duplicated genomic segments, or syntenic regions, which harbor large numbers of paralogous genes preserved from these ancient WGD events. We deleted four large, duplicated blocks, ranging from ~115 kb to ~684 kb using *Staphylococcus aureus* Cas9 to explore the effects of knocking out these blocks in *Arabidopsis*. Large deletions like these remain rare, especially in small and gene-dense plant genomes. Deletions were subsequently verified using whole-genome sequencing, which revealed minimal off-target effects. The number of deleted genes ranged from 16 to 60, and transposable elements ranged from 4 to 112 among the four deleted blocks. Two deletion lines showed distinct phenotypes resulting from the loss of many genes, while two others displayed no obvious defects, including for flowering time or hypocotyl elongation. Moreover, RNA-sequencing revealed that expression compensation, where deletions of paralogous genes lead to the upregulation of intact paralogues, was not a general response to the deleted regions under the conditions tested. Thus, it is possible to obtain viable plants when deleting large fragments that may be redundant or that contain nonessential genes. These results demonstrate that large chromosomal deletions can be used as a tool for genome engineering approaches, such as genome minimization in plants and allele replacement using homology-directed repair and other precision editing methods. Targeted deletions of large chromosome fragments will be a valuable tool for research and biotechnology applications.

## Significance

Plant genomes are shaped by ancient polyploidy events, leaving behind extensive duplicated regions whose biological roles remain largely uncharacterized. In this study, we demonstrate that targeted deletion of large, retained syntenic blocks in *Arabidopsis thaliana* results in viable plants, some with distinct phenotypes and widespread transcriptomic changes. These findings challenge the assumption that such regions are essential and highlight the potential redundancy or modularity within plant genomes. Our approach of removing entire duplicated blocks offers a powerful strategy to functionally dissect conserved genomic regions, investigate gene linkage and dosage effects, and accelerate the development of streamlined, minimal plant genomes. This work establishes a scalable framework for genome engineering with broad implications for plant biology, synthetic genomics, and biotechnology.

Flowering plants have undergone numerous whole-genome duplication (WGD) events (1–7), leading to the formation of a polyploid with multiple gene copies, or paralogues, which contribute to functional redundancy and expanded gene families. Over time, fractionation, the loss of DNA sequences following polyploidization, can return polyploid genomes to a diploid-like state (e.g., from tetraploid to diploid) (1–7). This process often results in the uneven retention or loss of duplicated chromosomal regions (homeologous regions) due to asymmetric fractionation, where one subgenome experiences more gene loss than the other. Duplicated genes may become subfunctionalized or neofunctionalized, contributing to genetic diversity and creating novel functions across paralogues (8–10). Despite plants being ideal for studying these effects due to their WGD history, the impacts of retained duplicated regions on plant traits following polyploidy events remain underexplored. Generating large deletions in these regions could help characterize their influence on phenotypes and inform strategies for genome minimization.

As a well-characterized model organism, *Arabidopsis thaliana* provides an ideal system for investigating the functional consequences of deleting duplicated genomic regions. Its genome is among the best annotated in plants, with a large number of genes experimentally validated, offering a robust framework for predicting phenotypic outcomes following gene loss. *Arabidopsis* has experienced three ancient polyploidy events defined as γ, β, and α, and has since returned to a diploid-like genomic state (1). Despite this, it retains 514 syntenic blocks, defined as regions of conserved gene collinearity that represent remnants of these ancient duplications (*Materials and Methods*). Each block corresponds to a homeologous region identified through collinear gene pairs. Importantly, genes essential for viability under laboratory conditions have been either experimentally identified or computationally predicted based on features associated with essential plant genes (11). This knowledge enables the strategic selection of syntenic regions with minimal or nonessential gene content, allowing us to generate deletions that are more likely to be viable while still providing insight into the evolutionary and functional significance of retained duplicated regions.

CRISPR/Cas9 systems have been widely adopted for efficient and precise genome editing across diverse organisms (12–17), including plants such as *Arabidopsis*, where deletions of varying sizes have been successfully generated (18–22). Large deletions have also been achieved in crops; for instance, a 245 kb deletion in rice (23) and a 1 Mb deletion in soybean (24), which demonstrates the scalability of CRISPR-based approaches for structural genome engineering. In this study, we employed a CRISPR/Cas9 strategy to delete duplicated genomic regions in *Arabidopsis* that were predicted to harbor fewer known lethal genes. We utilized the *Streptococcus pyogenes* Cas9 (SpCas9) orthologue *Staphylococcus aureus* (SaCas9). SaCas9 is smaller in size, recognizes distinct protospacer adjacent motif sequences, and functions with a unique guide RNA (gRNA) (25). It has been shown to work efficiently in both animal and plant systems (25, 26), and has been successfully used to mediate precise edits through homology-directed repair (HDR) and engineer large chromosomal rearrangements, such as inversions and translocations in plants (13, 26, 27). Therefore, we used SaCas9 to generate large deletions in *Arabidopsis* by deleting four syntenic genomic blocks of varying size. This allowed us to examine their functional impact and gain insight into the biological significance of retained duplicate regions derived from ancient polyploidy events in plant genomes.

## Results

### Identifying Syntenic Regions in *Arabidopsis* and Crops.

**Fig. 1.**
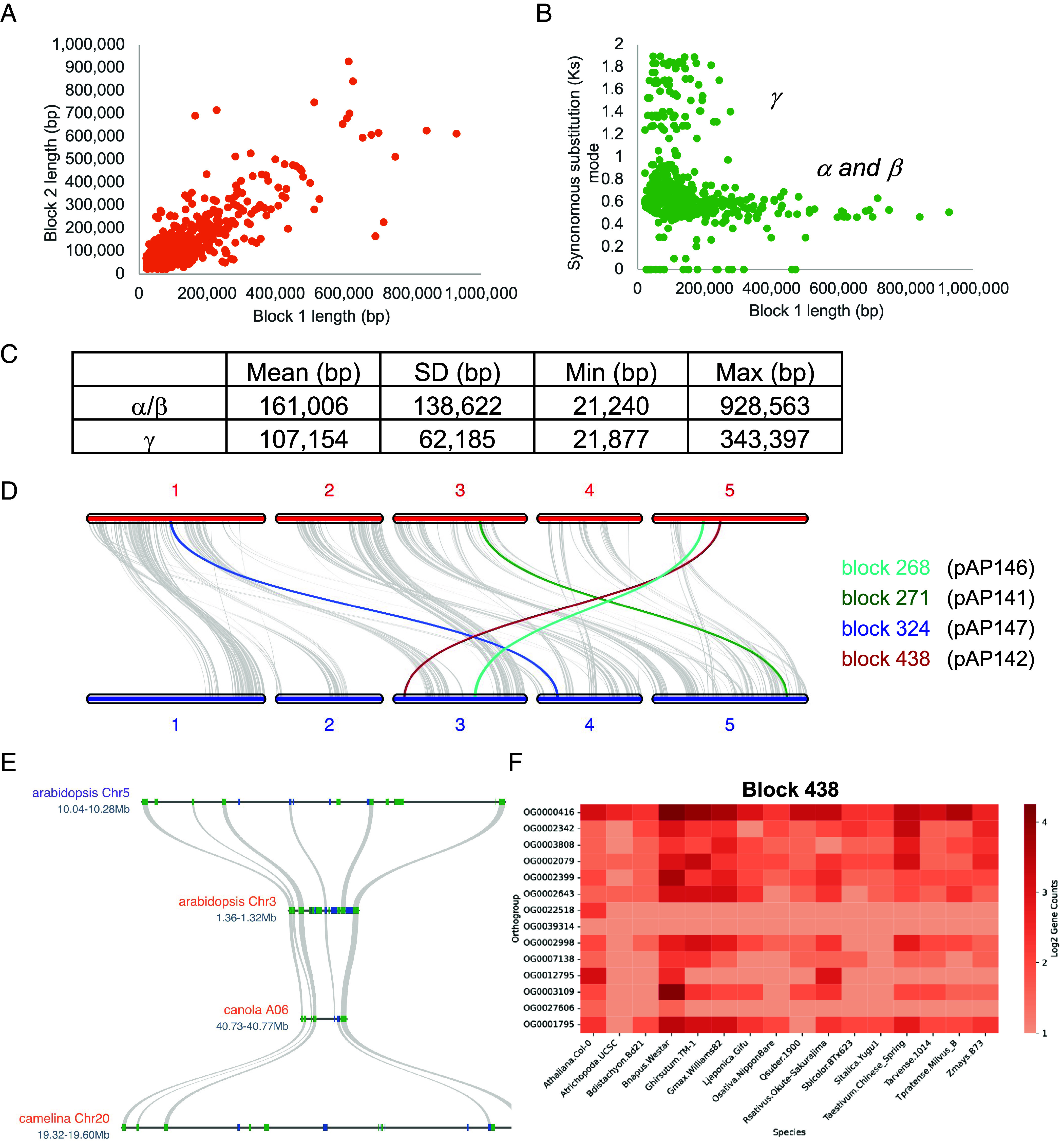
*Arabidopsis* syntenic blocks. (*A*) Scatterplot comparing syntenic block pairs across the *Arabidopsis* genome, which were defined as having at least five syntenic genes per 20 gene windows. (*B*) Synonymous substitution (Ks) rate was calculated for syntenic gene pairs and plotted as a function of block one length, which shows that blocks from the γ WGT are generally shorter since they are older than the α/β WGDs. The Ks mode was used to calculate duplication age. The age was then used to infer the polyploidy event from which the syntenic block arose. (*C*) Comparison of the syntenic block length between the γ WGT and α/β WGDs. (*D*) The four syntenic blocks across the *Arabidopsis* genome. Block 324 (blue ribbon), block 268 (cyan ribbon), block 271 (green ribbon), and block 438 (red ribbon); all syntenic blocks (gray ribbons). The constructs that were created to target each block are indicated in parentheses. (*E*) Comparison of block 438 with its pair (215) as well as one syntenic region in canola and camelina. (*F*) Heatmap representing the log-transformed (log_2_(count + 1)) gene counts across orthogroups (rows) and species (columns) for block 438. The color gradient indicates gene counts, where darker colors represent higher gene counts and lighter colors represent lower gene counts. Only orthogroups specific to the block were included.

*A. thaliana* has undergone a γ whole-genome triplication (WGT) followed by two more recent WGDs, β and α. Although the genome has returned to a diploid-like state, it retains large blocks of duplicated genes, or syntenic blocks, from these ancient polyploidy events (Fig. 1 *A*–*D* and Dataset S1) (1). We identified 514 syntenic blocks ranging in size from ~21 kb to ~929 kb, with relatively consistent distribution across the genome (Fig. 1*A* and Dataset S1). As expected, syntenic blocks from the older γ WGT are more highly fractionated, averaging ~107 kb [±62 kb (SD)], compared to ~161 kb [±139 kb (SD)] for the more recent α/β WGDs (Fig. 1 *B* and *C*). α/β blocks also retain more genes on average (43 genes) than γ blocks (26 genes), consistent with their higher conservation rates (~35% vs. ~29%). We selected four syntenic blocks from the α/β WGDs to explore the functional consequences of deleting retained duplicated regions. Candidate regions were chosen based on size and filtered to exclude known or predicted lethal genes (11), increasing the likelihood of recovering viable mutants. The selected blocks were 1) Block 271: ~124 kb on Chromosome 3 (30 genes), paired with ~54 kb on Chromosome 5 (22 genes); 2) Block 324: ~152 kb on Chromosome 4 (32 genes), paired with ~216 kb on Chromosome 1 (46 genes); 3) Block 438: ~251 kb on Chromosome 5 (16 genes), paired with ~49 kb on Chromosome 3 (17 genes); and 4) Block 268: ~691 kb on Chromosome 3 (61 genes), paired with ~164 kb on Chromosome 5 (37 genes) (Fig. 1*D* and Dataset S1). These syntenic regions vary in size across related Brassicaceae species. For example, block 438 is smaller in canola but expanded in camelina, highlighting the lineage-specific dynamics of genome fractionation (Fig. 1*E*).

While our initial analysis focused on genes retained within syntenic blocks, we recognized that not all duplicated genes remain associated with identifiable blocks. Extensive genome fractionation can disrupt the collinearity between homeologous regions, obscuring their syntenic relationships. Additionally, some genes may have been retained through tandem duplication or other duplication mechanisms (7). We analyzed orthogroups, gene families constructed across a panel of high-quality plant genomes, to capture this broader landscape of gene retention and potential redundancy. This approach allowed us to identify additional paralogues related to the genes within our deleted regions. Remarkably, some genes from the targeted blocks belong to orthogroups with over 40 members (Fig. 1*F* and *SI Appendix*, Figs. S1 and S2). These patterns of gene copy expansion in *Arabidopsis* were often mirrored across the broader plant genome panel, suggesting conserved evolutionary pressures on gene family expansion (Fig. 1*F* and *SI Appendix*, Figs. S1 and S2). The four syntenic regions selected for deletion, along with the number of genes and transposable elements (TEs) they contain, are summarized in Table 1. Detailed annotations, including gene functions, TE descriptions, and orthogroup membership, are provided in Dataset S2. Based on synteny and orthogroup analyses, we further categorized the genes in each deleted region as single-copy, syntenic paralogues, or members of multigene orthogroups (*SI Appendix*, Table S1). The majority of genes within these blocks possess at least one paralogue, either through synteny, tandem duplication, or orthogroup association, indicating that these regions may contain functionally redundant or dosage-sensitive genes. Their deletion offers a direct means to study the regulatory and phenotypic effects of removing retained duplicates, including potential gene dosage compensation, in which paralogues of deleted genes exhibit higher gene expression levels to mitigate effects resulting from deletions of duplicates.

**Table 1.** Deleted syntenic blocks

#	Block (#)	Chr (#)	Start (bp)	Stop (bp)	Length (bp)	Genes (#)	Tandem duplicates (#)	Lethal genes (#)	TEs/other annotations (#)	Syntenic genes (#)
1	271	3	16,628,704	16,753,190	124,486	27	19	0	8	7
1	488	5	24,228,178	24,282,157	53,979	15	5	2	1	7
2	438	5	10,030,493	10,281,880	251,387	16	3	0	34	7
2	215	3	1,318,096	1,367,508	49,412	17	0	3	0	7
3	268	3	14,954,587	15,645,446	690,859	60	6	0	112	11
3	425	5	7,002,453	7,166,904	164,451	29	2	4	10	11
4	324	4	6,415,287	6,567,629	152,342	32	14	0	4	12
4	66	1	11,858,251	12,073,833	215,582	46	13	2	12	12

Deleted syntenic blocks and their corresponding pairs. The first block in each of the four pairs was targeted for deletion. For blocks 271, 268, 438, and 324, the “Genes” column represents the number of genes deleted, and the “Genes” column for the corresponding block pairs represents the homeologous region of the deletion interval. The number of syntenic genes (CoGe) in each full block within a pair is equal since these syntenic paralogues define the regions of the paired blocks.

### Targeted Deletions of Four Large Syntenic Regions.

**Fig. 2.**
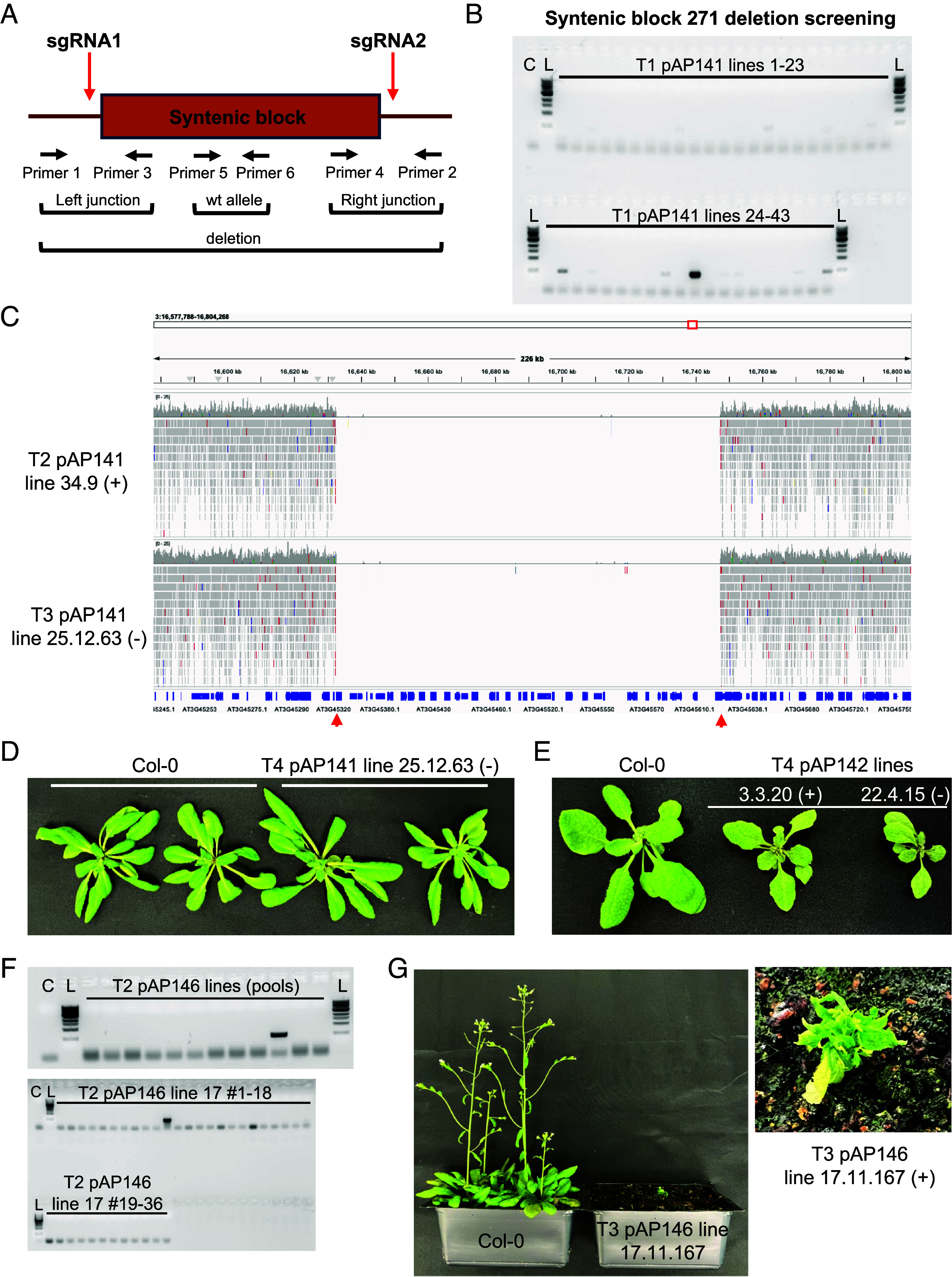
Deletion screening and characterization of deletion lines. (*A*) Schematic depicting the screening process to identify large deletions. Primers flanking gRNA sites were designed to detect whether a small PCR product can be obtained, signifying the presence of a deletion (deletion PCR, primers 1 + 2). Primers were also designed to amplify left and right junctions (primers 1 + 3 and 4 + 2, respectively), as well as to detect whether a wt allele is present in a sample (primers 5 + 6). (*B*) Gel displaying results from deletion PCR screening of 43 T1 pAP141 (construct targeting syntenic block 271) samples. C = Col-0, L = ladder. (*C*) Screenshot from the Integrative Genomics Viewer (IGV) showing whole-genome sequencing read alignments within syntenic block 271. Tracks from T2 and T3 samples are displayed. Red arrows represent designed gRNA spacers. (*D*) Two representative plants from Col-0 and a syntenic block 271 deletion line. (*E*) One representative Col-0 plant and plants from two independent syntenic block 438 deletion lines are displayed. (*F*) *Top* panel displays pooled screening of 12 pools of T2 pAP146 (construct targeting syntenic block 268) samples. The *Bottom* panel shows deletion PCRs of 36 individual plants from the positive pool. C = Col-0, L = ladder. (*G*) The first panel shows a comparison of Col-0 plants and a T3 homozygous line. The second panel shows a close-up view of a block 268 deletion line. “+” indicates samples containing the T-DNA, and “−” indicates null segregants.

We employed the SaCas9 system to generate large deletions, designing two gRNAs flanking each targeted syntenic region (Fig. 2*A*). Deletion events were identified using PCR primers located outside the gRNA target sites, combined with assays to detect the presence or absence of left and right junctions and the wild-type (wt) allele, allowing us to screen for homozygous deletion lines. The efficiency of each gRNA pair was confirmed by amplifying the targeted junctions and validating gRNA activity through Sanger sequencing (*SI Appendix*, Figs. S3 and S4). We targeted four retained duplicated regions derived from ancient WGD events. Each region was selected based on size, gene content, and the absence of known or predicted lethal genes (Table 1 and Dataset S2). The targeted deletions included 1) Block 271 (pAP141): ~115 kb, containing 27 genes and 8 TEs; 2) Block 324 (pAP147): ~151 kb, with 32 genes and 4 TE/other elements; 3) Block 438 (pAP142): ~250 kb, comprising 16 genes and 34 TE/other elements; and 4) Block 268 (pAP146): ~684 kb, harboring 60 genes and 112 TE/other elements. Most genes within these regions have no previously characterized phenotypes (Dataset S2), making them ideal candidates for exploring the functional consequences of deleting retained duplicated genomic segments.

In pAP141, initial screening of T1 plants identified a single deletion-positive line (line 34), verified by PCR and Sanger sequencing (Fig. 2*B* and *SI Appendix*, Fig. S5*A*). All T2 progeny from line 34 and progeny from an independent line (line 25) were homozygous for the deletion, with no amplification of wild-type junctions (Table 2 and *SI Appendix*, Fig. S5*B*). Whole-genome sequencing of these samples confirmed deletion integrity and specificity (Fig. 2*C* and *SI Appendix*, Fig. S6 *A* and *B*). Phenotypically, these lines were indistinguishable from Col-0 under laboratory conditions (Fig. 2*D* and *SI Appendix*, Fig. S6*C*), suggesting functional redundancy or tissue-specific phenotypic effects. In pAP147 lines targeting a ~151 kb deletion, several T2 plants showed deletion bands (*SI Appendix*, Fig. S7*A*). However, no homozygous plants were recovered from screening over 290 T3 individuals (*SI Appendix*, Fig. S7 *B* and *C*), despite confirmation of heterozygous deletions via Sanger sequencing and whole-genome analysis (*SI Appendix*, Figs. S7*D* and S8). The absence of homozygous samples suggests that essential gene(s) may reside within this region or that cumulative gene loss is lethal. For pAP142, seven of eight T2 pools (*Materials and Methods*) contained deletion-positive plants, and homozygous deletion lines were confirmed through PCR, Sanger sequencing, and whole-genome sequencing (*SI Appendix*, Figs. S9 and S10 *A* and *B*). Cas9-null segregants retained the deletion, indicating stable transmission. These lines displayed distinct phenotypes, including reduced size, serrated leaves, and pale green coloration, but were viable and fertile (Fig. 2*E* and *SI Appendix*, Fig. S10*C*). The observed phenotype was consistent with previously reported phenotypes (Dataset S2) for a mutant of a ribosomal gene (AT5G28060) (28, 29). Screening of 12 pooled T2 populations in pAP146 yielded one plant with the full ~684 kb deletion. PCR and sequencing confirmed junctions at expected gRNA sites (Fig. 2*F* and *SI Appendix*, Figs. S11 and S12*A*). Homozygous deletion plants exhibited severe developmental defects and sterility (Fig. 2*G* and *SI Appendix*, Fig. S12*B*). Despite the large gene loss, plants were viable under controlled conditions, with several observed traits matching known mutant phenotypes (e.g., AT3G42880, AT3G43210, AT3G43240) (Dataset S2).

**Table 2.** Deletion frequencies

Syntenic block	T2 line	Total plants screened	Number of plants with deletion
271	Line 25	34	6
271	Line 34	18	18
324	Line 1	44	1
324	Line 6	80	2
324	Line 17	77	4
438	Line 3	24	5
438	Line 13	14	2
438	Line 22	24	1
268	Line 17	36	1

Deletion frequencies of targeted syntenic blocks. The samples in this table [with the exception of line 34 (block 271) and line 17 (block 268)] represent single insertion lines. Lines targeting blocks 438 and 268 were initially screened in pools in the T2 generation. Individual plants from pools with deletion bands were subsequently screened to identify samples with deletions.

Off-target and phenotypic changes are potential consequences of our deletion strategy. We identified six potential off-target binding sites among the eight gRNAs used (*Materials and Methods* and Dataset S3). No evidence of editing was observed at these sites (*SI Appendix*, Figs. S13 and S14), supporting the specificity of SaCas9. Genome-wide variant analysis revealed minimal divergence from Col-0, except for pAP142 lines, potentially due to differences among Col-0 sublines or T-DNA transformation effects (*SI Appendix*, Fig. S15). Phenotypic assays, including hypocotyl length and flowering time, showed no significant differences for pAP141, pAP142, pAP146 (heterozygous), or pAP147 (heterozygous) lines compared to Col-0 controls (*SI Appendix*, Figs. S16 and S17). These results are consistent with the visual phenotypes observed in each line and suggest that dosage compensation or redundant gene activity may buffer major developmental impacts under standard growth conditions. Collectively, these results demonstrate that large, retained duplicated regions can be precisely and heritably deleted using SaCas9 in *Arabidopsis*. While some deletions show limited phenotypic impact, others result in visible developmental changes or lethality, highlighting the value of this approach for dissecting the functional consequences of ancient polyploidy events.

### RNA-seq Reveals Dosage Effects Resulting from the Deletion of Retained Duplicated Regions.

Differences in gene copy number and ploidy level may lead to dosage compensation and balanced expression levels (30–33). For example, certain paralogous genes can be silenced following WGD events to ensure expression levels remain at preduplication levels. Alternatively, it is also possible that retained paralogues may upregulate expression to compensate for mutated or deleted genes. In order to maintain balanced stoichiometric ratios, changes in expression of proteins and subunits in complexes may be coordinated following WGD. We performed bulk RNA-sequencing (RNA-seq) to investigate the transcriptional consequences of large-scale deletions in retained duplicated regions. We hypothesized that such deletions could influence global gene expression by either 1) altering the expression of paralogous genes to compensate for dosage loss or 2) perturbing regulatory networks connected to the deleted genes. We conducted bulk RNA-seq of pAP141 and pAP142 deletion lines, at two different time points in the morning and evening (*Materials and Methods*), in order to see whether time of day (TOD) impacted our ability to understand compensation in the deleted lines since greater than 90% of genes in *Arabidopsis* display peak expression a specific TOD (34). We also conducted RNA-seq of additional pAP142 plants that were sampled at a single time point and grown in different conditions (soil vs. plates) relative to the TOD samples (*Materials and Methods*). Additionally, we generated RNA-seq data for pAP146 wild-type, sibling controls, heterozygous, and homozygous deletion lines at a single time point to evaluate transcriptional effects across genotypes within this larger deletion background.

**Fig. 3.**
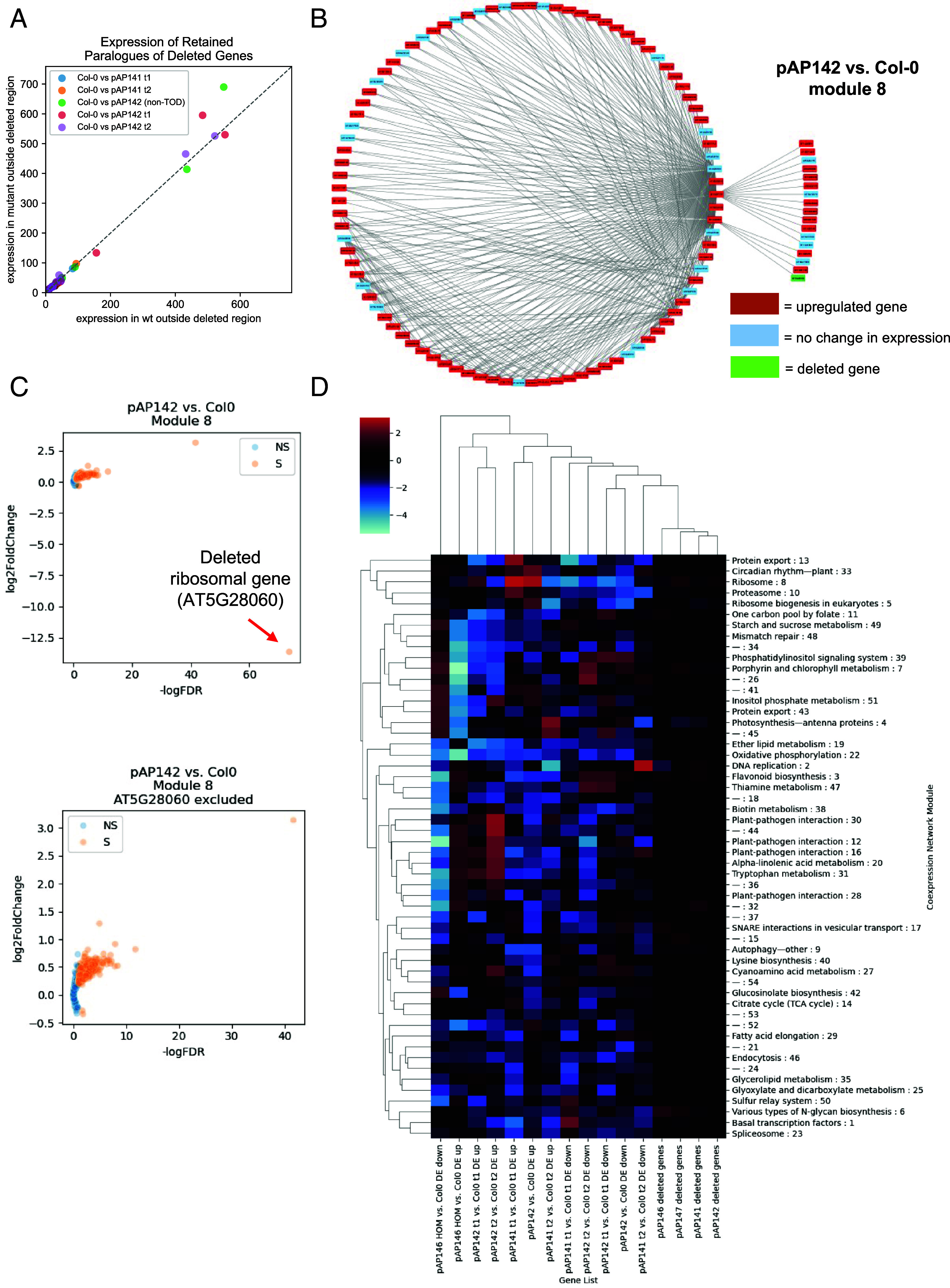
Investigation of compensation for deleted genes at the transcriptional level. (*A*) In Col-0 and the pAP141 and pAP142 deletion lines, total expression of the retained gene members of orthogroups affected by deletions shows that paralogues of deleted genes are generally not significantly affected. t1 = time point 1, t2 = time point 2. non-TOD refers to the separate set of pAP142 samples used for RNA-seq that were not part of the TOD experiment. (*B*) A network diagram representing a subset of module 8 from a large *Arabidopsis* coexpression analysis. Small network edges were filtered to a total network density of approximately 2.15 edges per gene. Any gene in the module that was left with no edges after this filtering was dropped from the visualization. This module contains a deleted ribosomal gene, AT5G28060, and is also strongly associated with our list of upregulated genes in that deletion line. (*C*, *Top*) A volcano plot showing significance of differential expression vs. change in expression for module 8 genes when comparing pAP142 deletion lines to Col-0. Module 8 genes are much more likely to be upregulated than we expect by chance. (*Bottom*) The same data as the *Top* panel, but with the deleted ribosomal gene, AT5G28060, removed to better visualize the other genes. (*D*) A heatmap representing associations between our gene lists of interest (deleted or DEGs) and coexpression network modules. The color reflects the log fold change between the observed intersection size of the module and gene list and the expected size assuming the two lists are independent.

Paralogous genes may retain ancestral functions or diverge through subfunctionalization or neofunctionalization (8–10). When a paralogue maintains the original or a related function, it can serve as a compensatory mechanism, preserving gene activity or expression in the absence of its counterpart and thereby buffering functional loss. Therefore, we examined whether deletion of a retained duplicate gene results in transcriptional dosage compensation by its paralogue. We explored both gene family analysis (orthogroups/paralogue groups) and synteny methods of identifying paralogues for our deleted genes. For each method, we identified paralogous gene groups and evaluated the expression of retained paralogues of deleted genes to assess whether wt expression levels were maintained through compensatory transcriptional regulation. For each of our sample sets, we measured total expression levels of genes within paralogue groups that were outside of deleted regions and genes within deleted regions in wt and mutant lines (Fig. 3*A* and *SI Appendix*, Figs. S18–S20). If there were dosage compensation in our lines, we expect the total expression of paralogue groups, when only considering genes outside deleted regions, to increase in the mutant populations to retain overall wt expression levels. However, we did not observe such changes in our pAP141 and pAP142 lines when compared to Col-0 across the two collected timepoints. Because group-level analysis may obfuscate important differences in individual genes, we also looked at differential expression in each syntenic paralogue of a deleted gene in Col-0 and deletion lines and found only one marginal case of a retained paralogue significantly increasing in expression in response to the deletion event (*SI Appendix*, Figs. S21–S23 and Datasets S2 and S4). A single ribosomal gene (AT3G04920, ES24Z) displayed increased expression in the pAP142 lines, where its paralogue (AT5G28060, ES24Y) was deleted. Notably, this small but significant increase in expression of the paralogue was present in pAP142 time point 1 samples, which were grown on plates, and in the non-TOD pAP142 samples, which were grown in soil (*Materials and Methods*). However, we observed previously observed phenotypes associated with AT5G28060 mutants (28, 29), indicating that any increase in paralogue expression was not sufficient to compensate for the loss of this gene. These results indicate that paralogues of these deleted genes do not generally compensate for the loss of a paralogue via direct changes in expression.

We next explored pAP146 heterozygous and homozygous lines to determine whether there are any dosage compensation effects resulting from the deletion of this large region. The total expression of several orthogroups of genes outside the deleted region increases in the homozygous deletion line, suggesting the presence of possible compensation mechanisms (*SI Appendix*, Figs. S24 and S25). However, since homozygous lines display severe developmental phenotypes, it is difficult to interpret whether there is true dosage compensation or whether the differences in expression are a result of different developmental stages (*Materials and Methods*). There was no detectable expression of orthogroups when considering genes within deleted regions in pAP146 homozygous lines compared to Col-0 or wt sibling controls, as expected. For heterozygous lines, we observed that total orthogroup expression within the deleted region was approximately half of what was observed in wt samples, indicating that there is a linear relationship between copy number and expression for these genes (*SI Appendix*, Fig. S24*B*). Consistent with the orthogroup analysis, expression levels of retained syntenic paralogues of deleted genes exhibited similar trends in pAP146 samples (*SI Appendix*, Figs. S26 and S27 and Dataset S2).

Dosage compensation can extend beyond individual gene pairs to the level of gene regulatory networks, where the loss of a single gene may induce broader transcriptional shifts to maintain cellular homeostasis. To explore this possibility, we utilized a previously published *Arabidopsis* coexpression network comprising over fifty modules (35) to assess how the deleted genes in our four targeted regions were distributed across coexpression modules. Genes from the deleted regions were broadly dispersed among many modules, with no significant enrichment in any single module (Dataset S5), suggesting that the deletions affected a wide range of biological processes rather than disrupting specific functional clusters. In addition, genes within deleted regions were characterized as having average centrality, indicating that there is no significant difference when compared to a random set of genes (*Materials and Methods* and *SI Appendix*, Table S2). We next analyzed whether differentially expressed genes (DEGs) in our deletion lines were enriched within particular modules, allowing us to explore the transcriptional consequences of such deletions and reveal possible evidence of coordinated compensatory responses at the network level. Indeed, we observed significant enrichment of up- and downregulated DEGs in distinct modules across several deletion lines (Dataset S5). Notably, genes upregulated in pAP142 lines were highly enriched in module 8, which is annotated for translation-related processes (35) (Dataset S5). A network diagram of a subset of genes present in module 8, as well as plots depicting differential expression of all module 8 genes, display this upregulation within the module (Fig. 3 *B* and *C*). DEGs from the other deletion lines were also enriched across various modules (Fig. 3*D*). One of the genes deleted in pAP142, AT5G28060 (ES24Y), a ribosomal protein gene, has a paralogous gene that also showed evidence of dosage compensation (*SI Appendix*, Figs. S20*B* and S23*B*). The concurrent upregulation of translation-associated genes within this network module, alongside the marginal compensatory expression of the paralogue, suggests a multilayered transcriptional response to maintain translation capacity following gene loss. This finding highlights how network-level compensation may complement or reinforce gene-level dosage effects, particularly for genes involved in essential biological processes such as protein synthesis. Future analyses of networks in wt and deletion lines will elucidate whether upregulation of genes and connectivity changes coordinate a network-level dosage response.

We identified numerous additional examples of upregulated DEGs in deletion lines that were significantly enriched, often more than fourfold, within specific coexpression modules (Fig. 3 *B*–*D*, *SI Appendix*, Fig. S28, and Dataset S5). These modules were annotated with diverse molecular functions, including phosphorelay response regulation, kinase activity, adenosine diphosphate binding, transcriptional regulation, and ligand-gated ion channel activity. Notably, several of these gene categories are known to be preferentially retained following WGD events and may therefore contribute to dosage compensation mechanisms (3). Conversely, downregulated DEGs in deletion lines were also enriched within distinct modules (Fig. 3*D*, *SI Appendix*, Fig. S29, and Dataset S5). These were annotated with functions such as nucleic acid binding, microtubule binding, catalytic activity, and DNA supercoiling activity, some of which are associated with highly conserved genes that tend to be overfractionated following WGD (3). Rather than compensating for gene loss, downregulation of these genes may reflect dosage balance adjustments, where the loss of one gene necessitates decreased expression of functionally linked partners to maintain stoichiometric balance within essential biological pathways. We observed transcriptional consequences resulting from the deletions of many genes. Thus, to further explore whether the enrichment of some DEGs in certain modules corresponds to network-level dosage compensation, additional research is needed, such as proteomics studies and network reconstruction in deletion lines.

We next investigated expression changes of neighbors of deleted genes. A logistic regression was performed to determine whether the strength of a gene’s connection to a deleted gene can be used to predict whether a particular gene was differentially expressed (*Materials and Methods* and Table 3). Genes that are strongly connected to deleted genes within the coexpression network are significantly more likely to be downregulated in pAP146 and pAP141 lines and less likely to be upregulated in pAP146, pAP141, and pAP142 t2 samples (Table 3). This may result from possible gene dosage balance adjustment. No significant relationship was observed for downregulated genes in all pAP142 conditions tested, suggesting a distinct mode of regulation for these samples. However, for pAP142 t1 and non-TOD samples, genes that are strongly connected to deleted genes are significantly more likely to be upregulated, which suggests a possible expression compensation mechanism. It is noteworthy that we observed this phenomenon in pAP142 t1 and non-TOD samples since we also observed a slight increase in expression of the paralogue (AT3G04920, ES24Z) of a deleted ribosomal gene (AT5G28060, ES24Y), and enrichment of upregulated DEGs from pAP142 t1 and non-TOD samples within a translation-related module (Datasets S2 and S5). To determine whether this deleted ribosomal gene may be contributing to our observed results, we repeated the logistic regression analysis for pAP142 lines after excluding AT5G28060 from our network graph. Strikingly, genes with strong connections to deleted genes were now more likely to be downregulated in pAP142 t1 and non-TOD samples, further providing evidence for a possible compensation mechanism due to the deletion of AT5G28060 (Table 3).

**Table 3.** Association between coexpression network connectivity to deleted genes and differential expression status across lines

Deletion	Comparison	Coef.	pval	PR2	FDR	Significant
pAP146	pAP146 hom vs. Col0 DE up	−14.857872	2.04E-52	0.00962156	6.11E-52	TRUE
pAP146	pAP146 hom vs. Col0 DE down	24.2881666	5.23E-169	0.03322183	9.41E-168	TRUE
pAP142	pAP142 vs. Col0 DE up	30.0028576	3.82E-98	0.03944347	3.43E-97	TRUE
pAP142	pAP142 vs. Col0 DE down	3.46350264	0.12478566	0.00024744	0.13212599	FALSE
pAP142	pAP142 t1 vs. Col0 t1 DE up	12.7197046	2.71E-22	0.00508699	4.88E-22	TRUE
pAP142	pAP142 t1 vs. Col0 t1 DE down	2.58669545	0.11521447	0.0001541	0.12961628	FALSE
pAP142	pAP142 t2 vs. Col0 t2 DE up	−166.60818	3.37E-55	0.04512803	1.52E-54	TRUE
pAP142	pAP142 t2 vs. Col0 t2 DE down	2.51749235	0.27446675	0.00012593	0.27446675	FALSE
pAP141	pAP141 t1 vs. Col0 t1 DE up	−34.989581	3.18E-08	0.00504162	3.82E-08	TRUE
pAP141	pAP141 t1 vs. Col0 t1 DE down	17.9506571	7.76E-18	0.0054453	1.27E-17	TRUE
pAP141	pAP141 t2 vs. Col0 t2 DE up	−536.88586	2.95E-32	0.06384254	6.64E-32	TRUE
pAP141	pAP141 t2 vs. Col0 t2 DE down	40.5771029	5.77E-58	0.05077108	3.46E-57	TRUE
pAP142 (-AT5G28060)	pAP142 vs. Col0 DE up	−37.176696	1.70E-11	0.00588706	2.35E-11	TRUE
pAP142 (-AT5G28060)	pAP142 vs. Col0 DE down	20.3972096	1.15E-12	0.00477154	1.73E-12	TRUE
pAP142 (-AT5G28060)	pAP142 t1 vs. Col0 t1 DE up	−98.231398	1.10E-49	0.02444845	2.84E-49	TRUE
pAP142 (-AT5G28060)	pAP142 t1 vs. Col0 t1 DE down	21.3314313	1.47E-23	0.00571202	2.94E-23	TRUE
pAP142 (-AT5G28060)	pAP142 t2 vs. Col0 t2 DE up	−192.34296	3.18E-53	0.04179657	1.14E-52	TRUE
pAP142 (-AT5G28060)	pAP142 t2 vs. Col0 t2 DE down	17.6761642	2.02E-09	0.0034081	2.60E-09	TRUE

For each deletion line and comparison, a logistic regression was used to test whether the strength of a gene’s connection to the deleted genes (defined as the maximum edge weight to any deleted gene) predicted whether that gene was differentially expressed. “Deletion” and “Comparison” columns specify the deletion line and the differential expression comparison used. For samples with “-AT5G28060” labels, AT5G28060 was excluded from the analysis. “Coef.” gives the logistic regression coefficient, indicating the direction and magnitude of the association. “PR2” reports McFadden’s pseudo-R^2^ as a measure of model fit. “pval,” “FDR,” and “Significant” indicate the raw *P*-value, the Benjamini–Hochberg adjusted *P*-value, and whether the association was significant at a 5% FDR threshold.

Together, these findings illustrate that gene expression responses to large-scale deletions are modular, context-dependent, and shaped by evolutionary constraints on gene retention and loss. By integrating orthogroup relationships, syntenic paralogue expression, and network-level analyses, we provide a comprehensive view of the global transcriptomic consequences of deleting retained duplicated regions in the *Arabidopsis* genome.

## Discussion

In this study, we demonstrated that large, retained duplicated regions in the *Arabidopsis* genome can be precisely and heritably deleted. By targeting four syntenic blocks selected for the absence of known or predicted lethal genes (11), we successfully generated homozygous deletion lines for three blocks and heterozygous lines for the fourth. The inability to recover homozygous deletions for the fourth region suggests the presence of uncharacterized essential genes, loss of redundant paralogues with synthetic lethality, or divergence through neofunctionalization or subfunctionalization (30). It is noteworthy that we recovered a viable homozygous line with a ~684 kb deletion encompassing ~60 genes and 112 TEs, confirming the feasibility of engineering large chromosomal deletions.

Through deletion of entire syntenic blocks, we directly assessed the functional impact of removing groups of retained duplicate genes. Phenotypic outcomes ranged from no observable differences to severe developmental defects, highlighting the variable consequences of large-scale gene loss. In several cases, plants exhibited clear morphological phenotypes (Fig. 2 *E* and *G*), while key traits such as hypocotyl length and flowering time remained largely unchanged (*SI Appendix*, Figs. S16 and S17), suggesting that some developmental processes are more robust to gene dosage changes than others. Although we found no strong evidence for transcriptional dosage compensation in the pAP141 and pAP142 deletion lines, it is possible that compensation occurs at the orthogroup or syntenic paralogue level in specific tissues and cell types not captured by our bulk RNA-seq data. In addition, compensation may also occur at distinct spatiotemporal contexts in mutant lines resulting from the chromatin contexts in which paralogues are located. Growth conditions may also play a factor, in which compensation may occur in specific contexts, but not others (e.g., plants grown on plates vs. soil). Multiple generations may also be required before expression compensation mechanisms begin to react to deletions.

We observed a small but significant increase in the expression of a paralogue of a deleted ribosomal gene [AT5G28060 (ES24Y)], consistent with previous reports of gene dosage compensation following the deletion of ribosomal RNA genes in *Arabidopsis* (36). However, significant differences in paralogue expression were observed in the pAP142 t1 and non-TOD samples, but not in pAP142 t2. In contrast, the pAP146 homozygous lines showed signs of potential compensatory expression, though interpretation is complicated by developmental stage and tissue-specific differences at the time of sampling (*Materials and Methods*). Importantly, orthogroup expression levels in heterozygous lines were reduced by approximately 50%, providing evidence for a direct correlation between gene copy number and transcript abundance. Together, these results demonstrate that large-scale deletions can be a powerful approach for investigating the roles of retained duplicates and the dynamics of dosage-sensitive gene networks. In some cases, compensatory mechanisms, likely operating at the transcriptional or network level, may buffer the effects of gene loss, enabling viability despite substantial genomic deletions.

**Fig. 4.**
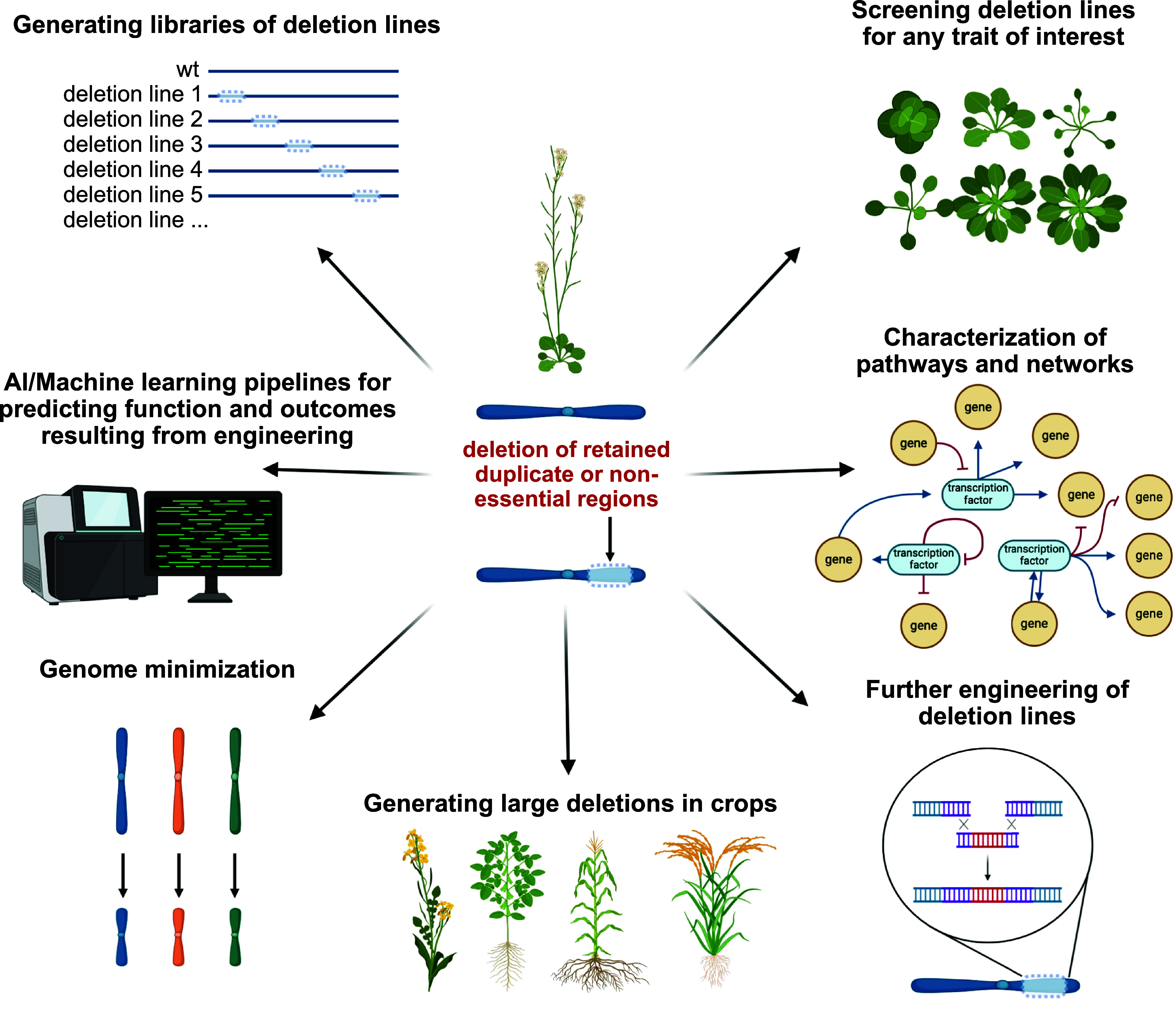
Generation of deletion lines (deletions of retained duplicate regions or nonlethal genomic segments) and their applications. Libraries of large deletion lines in plants (analogous to a T-DNA population) can be generated, such as in *A. thaliana* or crop species, and screened for traits of interest, subsequently leading to the characterization of genes with unknown functions. Furthermore, novel pathways and networks can be discovered through our approach and further elucidate the significance of duplicated regions that have been retained. Deleted genomic segments within various lines can also serve as landing pads for precision engineering methods (e.g., Cas9-mediated HDR or other precision methods). Deletion of syntenic regions and/or nonessential segments of genomes provides a path toward eukaryotic genome minimization. By integrating genomics data (such as single-nuclei RNA-seq) and phenotypic information from a library of deletion lines, AI/machine learning pipelines can be utilized to predict outcomes resulting from new deletions and to predict functions of unknown genes and uncharacterized noncoding genomic regions. Figure created with BioRender.com.

Because the process of genome fractionation reflects both evolutionary selection and random genetic drift, retained duplicated regions represent valuable targets for both biotechnology and basic research applications. These include the development of deletion line libraries, genome minimization (targeting both genes and TEs) as has been done in bacteria and yeast (37, 38), precision genome engineering, and testing emerging hypotheses such as the genetic basis of heterosis (Fig. 4). Syntenic block deletions offer a powerful tool for exploring gene function, redundancy, and viability in plant genomes. Similar to transferred DNA (T-DNA) mutant collections, populations of plants harboring defined syntenic deletions could serve as screening platforms for phenotypic analysis. Observed phenotypes could then be mapped to individual genes within the deleted regions, enabling functional dissection of multigene blocks. This approach also lays the groundwork for systematic genome minimization, whereby large genomic segments are sequentially or combinatorially deleted to identify the minimal gene set required for plant development, fitness, and adaptation. In addition to our approach, by considering and integrating the centrality of target genes, a higher proportion of viable plants may be obtained when deleting large genomic fragments characterized by genes with low to average centrality. Such deletions may uncover hierarchies within regulatory networks and reveal core components of essential biological pathways.

This strategy also supports advances in precision genome engineering. Deleted chromosomal regions can act as landing pads for HDR and other precision editing methods, allowing the targeted insertion of new sequences. Given the generally low efficiency of HDR in plants, especially for large fragment replacements, a two-step strategy that first deletes a region and then replaces it may enhance editing success rates. The utility of large-scale deletions is not limited to *Arabidopsis*. In plant species with lower gene density or high repeat content, such as maize (4), larger deletions may be more easily tolerated due to the reduced likelihood of removing essential genes. Polyploid species, in particular, retain extensive genomic redundancy and may be especially amenable to the removal of large chromosomal segments without compromising viability.

Deletions spanning kilobase to megabase scales provide a versatile means to study plant development, environmental adaptation, and phenotypic diversity. Beyond functional genomics, this approach may facilitate synthetic fractionation of conserved genomic regions or deletions of nonessential regions, offering a lens to dissect regulatory architecture and uncover the molecular underpinnings of heterosis. For example, by crossing deletion lines derived from distinct genetic backgrounds and evaluating hybrid performance, it may be possible to map loci contributing to heterosis and yield enhancement. In summary, targeted deletion of syntenic and nonsyntenic genomic regions represents a flexible and scalable platform for both fundamental plant biology and applied biotechnology, with potential applications in gene function discovery, genome streamlining, and crop improvement.

## Materials and Methods

### Plant Material, Selection, and Screening.

The Col-0 ecotype of *A. thaliana* was used for this study. Plants were grown in long-day conditions at a constant temperature of 23 °C. To generate transgenic plants, GV3101 agrobacteria (Intact Genomics) were used for the floral dip method (39). T1 plants were screened on ½ MS plates containing BASTA (0.9% Phytoagar, ½ Murashige and Skoog Basal Medium, 20 μg/mL BASTA) to identify transgenic lines.

Genomic DNA was extracted from plants individually or in pools using an adapted version of the Edwards et al. protocol (40). The DNeasy 96 Plant Kit (Qiagen) was also used for high-throughput genomic DNA extractions. T1 and T2 lines were genotyped to determine whether deletions of syntenic regions were induced. Deletion PCRs, wt allele PCRs, left and right junction PCRs, and T-DNA genotyping PCRs were then run to characterize lines. For pAP141 and pAP147 lines, plants were screened individually. For pAP142 and pAP146 lines, plants were initially screened in pools. Leaf tissue from T2 pAP142 lines was initially pooled into eight different tubes (with 18 to 25 plants per tube), and deletion PCRs were run to find pools containing deletion bands. Individual plants from positive pools were then screened to identify samples with deletions. wt allele PCRs were also run to find homozygous plants. Similarly, 12 pools (with up to 48 plants per pool) were screened by deletion PCRs to identify deletions in T2 pAP146 lines. One pool containing a potential deletion was found, and subsequent screening of individual plants showed that there was 1 plant containing a deletion.

### Vector Design and Cloning.

Constructs used in this study were cloned into the pDe_SaCas9 vector containing an egg cell-specific promoter driving the expression of SaCas9 (26, 27). gRNA spacer sequences were designed using CRISPRdirect (41). Spacers were designed to be 21 nucleotides (nt) in length, as it has been shown previously that 21 nt spacers work well for SaCas9 (25, 26). 20 nt complementary oligos corresponding to each of the spacers and containing 4 bp Golden Gate overhangs were annealed, and subsequently individually Golden Gate assembled into the pEn_Sa_Chimera vector (26) using BbsI, creating 8 entry vectors. Since the entry vector already contains a U6 promoter to drive the expression of the gRNA cassette, the initial “G” transcribed from this promoter is included in the spacer sequence, thus creating 21 nt spacers. Each spacer was designed in such a way so that the 21 nt is fully complementary to the genomic target site.

To create the vector pAP141, pDe_SaCas9 was initially digested with MluI. Then, the sgRNA2 cassette was amplified from the entry vector with primers containing homology arms for Gibson assembly. The amplicon was then purified and Gibson assembled with the MluI digested vector. The sgRNA1 cassette was then added via Gateway cloning to create the final vector. Vectors pAP142, pAP146, and pAP147 were created in a similar manner.

### Identification of Syntenic Blocks and Synteny Analysis.

Syntenic blocks in the *A. thaliana* genome (TAIR10) were identified using the SynMap tool on the Comparative Genomics (CoGe) (42) platform using specific parameters (DagChainer: -D 20, -A 5; Merge Syntenic Blocks: Quota Align Merge; CodeML: Synonymous Ks). Tandem duplicated (TD) genes were also determined as part of the analysis. The resulting final syntenic gene-set output file was parsed using a custom script (Dataset S1). Python scripts were developed to calculate the number of duplications for each *Arabidopsis* gene within syntenic block regions, as well as the number of syntenic blocks containing each deleted syntenic gene (Dataset S1). Syntenic blocks were visualized and checked using MCscan Python version (v 1.3.3) using the same settings as SynMap in CoGe; TDs were identified with default settings. Comparisons with canola and camelina were carried out with genomes downloaded from BnaOmics (https://bnaomics.ocri-genomics.net/organism/10327; westar.v0) and EnsemblPlants (https://plants.ensembl.org/Camelina_sativa/Info/Annotation/, GCA_000633955.1), respectively.

### Orthogroup and Gene Ontology (GO) Analysis.

To classify *Arabidopsis* genes into orthogroups, Orthofinder (v 2.5.4) with “-m MSA” was used. This analysis was conducted using a curated dataset of gold-standard flowering plant reference genomes sourced from reputable databases such as Phytozome and Lotus BASE, representing a diverse range of species from both monocots and dicots. The monocots include *Oryza sativa* (NipponBare, Phytozome v7), *Zea mays* (B73, Phytozome RefGen_V4), and *Sorghum bicolor* (BTx623, Phytozome v3.1.1). The dicots include *Glycine max* (Williams82, Phytozome Wm82.a4.v1), *Brassica napus* (Westarv0, BnPIR), and *A. thaliana* (Col-0, Phytozome Araport11). Only the primary protein models were included, defined as the longest CDS unless otherwise specified by the source.

Gene counts across orthogroups and species for each deleted syntenic block were analyzed by parsing Orthofinder results using the deleted *Arabidopsis* genes. Counts for Amborella and the representative monocots and dicots were log-transformed (log_2_(count + 1)). Heatmaps of log-transformed gene counts were generated using seaborn (v 0.12.2) in Python to visualize variation across species. Functional annotations were generated using eggNOG-mapper (v 2.1.12). For each deleted syntenic block, GO terms, Kyoto Encyclopedia of Genes and Genomes annotations, and orthogroups were compiled for the corresponding genes (Dataset S2).

### Whole-Genome Sequencing and Analysis.

For whole-genome sequencing, genomic DNA was initially extracted using the DNeasy Plant Mini Kit (Qiagen). Illumina DNA libraries were then prepared (Illumina DNA Prep), and paired-end 150 bp reads were obtained by sequencing on the Illumina NextSeq2000 platform. The reads were first controlled for quality using FastQC v0.11.9. In order to verify the locations of the deletions in each sample, the reads were then mapped to the Araport11 *A. thaliana* Col-0 genome from Phytozome (https://phytozome-next.jgi.doe.gov/info/Athaliana_Araport11) using minimap2 v2.24-r1122 (43) and samtools v1.17 (44) to sort the resulting mapping file.

### Off-Target Analyses.

Predicted off-target binding sites for the SaCas9 gRNAs were identified using Cas-OFFinder (45). As parameters for identifying off-target sites, a mismatch number equal to or less than three, DNA bulge size equal to or less than two, and RNA bulge size equal to or less than one were used. Global off-target structural variant analysis was conducted by running the short-read SV caller smoove v0.2.8 on the genomic mapping files and the Araport11 genome annotations to produce an annotated variant call format file. The VCF file was then filtered for calls with a Phred quality score greater than or equal to 20. The number of sites that were called as homozygous for the reference was counted for all deletion samples and *A. thaliana* Col-0 control samples. Statistical significance between each deletion sample group and the control group was calculated using Dunnett’s Test.

### Flowering Time and Hypocotyl Assays.

For flowering time assays, plants were grown in long-day conditions at a constant temperature of 21 °C. For measuring hypocotyl lengths, seeds were plated on ½ MS plates. Plates were then wrapped with aluminum foil and placed in 4 °C for at least 3 d for stratification. They were then placed in a growth chamber and grown vertically for 5 to 7 d in the dark (wrapped in aluminum foil) at a constant temperature of 21 °C. Plates were then scanned, and hypocotyl lengths of the deletion lines were quantified using ImageJ.

### RNA Extractions and Library Preparation.

Samples for RNA-seq were grown in long-day conditions at a constant temperature of 21 °C. For pAP141 and pAP142 lines, seeds were plated on ½ MS plates along with Col-0 controls. After ~2 wk of growth, seedlings were collected at two different time points [evening (t1) and morning (t2)] for RNA extractions. Several replicates, each consisting of pooled seedlings (~8 to 10), were included. We also conducted RNA-seq of an independent set of pAP142 samples, where individual seedlings were collected for RNA extraction after ~2 wk of growth in soil, along with Col-0 controls. For pAP146 lines, leaves from individual plants were collected from Col-0, sibling controls, and heterozygous samples after ~2 wk of growth. For pAP146 homozygous lines, seedlings were collected for RNA extraction since these lines were delayed developmentally. All RNA extractions were done using the Direct-zol RNA Miniprep Kit (Zymo Research). To prepare libraries for RNA-seq, the Illumina Stranded mRNA Library Prep Kit was used. Libraries were sequenced on the NovaSeqX platform (10B flowcell, 100 cycles).

### RNA-seq Analyses.

RNA-seq libraries were mapped to the Araport11 (46) reference genome using hisat2(v2.2.1) (47). Those alignments were visually inspected using JBrowse2 to verify the absence of expression in homozygous mutants indicative of successful deletions. The RNA-seq libraries were also quantified against the Araport11 primary transcriptome using salmon(v1.6.0) (48). The resulting counts were then imported into DESeq2(v1.44.0) (49) with design formulae relevant to each experiment. The design formula for pAP142, pAP146, and pAP147 was genotype, while the TOD formula utilized a combination of genotype and time point. Normalization was then performed on the counts using DESeq2 to estimate size factors via the median ratio method. Differential expression analysis was then conducted with DESeq2, modeling the normalized count data with a Negative Binomial distribution. Statistical contrasts were extracted from the DESeq2 output with the “results” command to compare all relevant experimental groups, with control samples serving as the baseline when applicable. Results from DESeq2 are provided in Dataset S4.

In order to investigate potential gene compensation effects, we used the previously described Orthofinder results to identify paralogues of our deleted genes within *Arabidopsis*. If compensation at the expression level is occurring, we should expect the expression of paralogues outside the deletions to go up in the homozygous mutant lines compared to the wt controls to compensate for the loss of expression in the deleted region. For each gene in a deleted region, we identified the orthogroup it belonged in, then computed the total expression of genes inside the deleted region in that orthogroup and outside the deleted region in that orthogroup. We could then directly compare the expression of the paralogues of deleted genes (the genes in the orthogroup but outside the deleted region) between the wt and deletion lines, as well as confirm the loss of expression in the deleted region. This analysis was repeated using syntenic gene groups defined by MCscan results which produced smaller groups. Because this analysis may obfuscate effects specific to a single paralogue within a larger group of paralogues, we also looked directly at the paralogues of deleted genes individually using our prior described differential expression results.

To further elucidate potential compensation mechanisms and characterize our deleted genes and our DEGs, we accessed a large public *Arabidopsis* gene coexpression network (35). This network categorized genes into 54 modules. We conducted a hypergeometric test to evaluate whether any of these modules were associated with our deleted regions or the sets of DEGs in our deletion lines. We applied a Benjamini–Hochberg correction and reported all associations with a corrected *P*-value below 0.05 as significant. We also assessed the centrality of deleted genes using Weighted Network Connectivity (WNC), a measure of gene centrality in the coexpression network (50). For each deleted region, we calculated the mean WNC of the genes within that region and compared it to a null distribution generated from 10,000 bootstrap samples, each consisting of a randomly selected gene set of equal size. To estimate a *P*-value, we used a two-tailed test based on the absolute difference between the observed mean WNC and the bootstrap distribution mean—that is, the *P*-value reflects the proportion of bootstrap samples with a more extreme absolute deviation from the mean than that observed for the deleted region. We also tested whether proximity in the graph to deleted genes was predictive of differential expression status. For each nondeleted gene, we identified the strongest weight connecting that gene to any of the deleted genes, and then used those weights to predict differential expression status using a logistic regression model. Upon seeing that strong connections to pAP142 deleted genes were associated with upregulation, we suspected that activity in module 8 via AT5G28060 may be explaining the results. Thus, we repeated the analysis after excluding AT5G28060 from the graph. In this subsequent analysis, we found that strong connections to deleted genes were now associated with downregulation.

## Supplementary Material

Appendix 01 (PDF)

Dataset S01 (XLSX)

Dataset S02 (XLSX)

Dataset S03 (XLSX)

Dataset S04 (XLSX)

Dataset S05 (XLSX)

## Data Availability

Custom code/scripts used in this study have been deposited in GitLab (https://gitlab.com/salk-tm/syntenic_deletions_project) (51). Whole-genome sequencing and RNA-sequencing data have been deposited in the Sequence Read Archive (SRA) and Gene Expression Omnibus (GEO) databases and can be accessed with the accession numbers PRJNA1210642 (52) and GSE287593 (53), respectively.
