# Supplementary material for: Targeted deletions of large syntenic regions in Arabidopsis thaliana
Source: Proc Natl Acad Sci U S A. 2025 Aug 11;122(33):e2419744122. doi: 10.1073/pnas.2419744122 (PMC12377758; doi:10.1073/pnas.2419744122)
Supplement: Supplementary file 1 — Appendix 01 (PDF) [file pnas.2419744122.sapp.pdf]

PNAS

Supporting Information for:

Targeted deletions of large syntenic regions in *Arabidopsis thaliana*

Ashot Papikian[a], Rachel J. Rattner[a], Jenni Kao[a], Neil Hauser[a], Nicholas Allsing[a], Allen Mamerto[a], Nolan T. Hartwick[a], Kelly Colt[a], Todd P. Michael[a,b,c,d,1]

Corresponding author: Todd P. Michael
Email: tmichael@salk.edu

This PDF file includes:

Figures S1-S29, Tables S1-S2

Other supporting materials for this manuscript include the following:

Datasets S1-S5

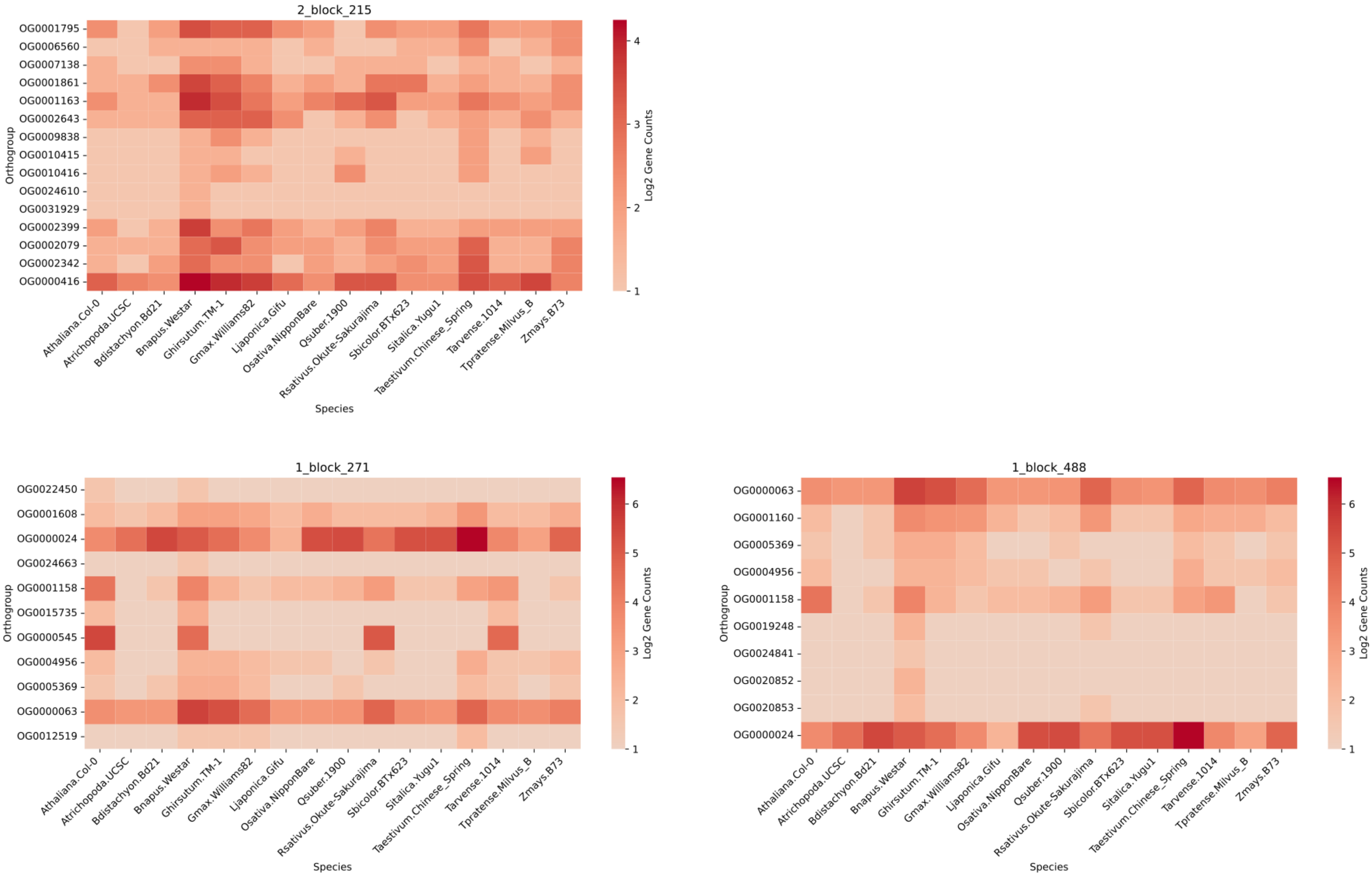

Fig. S1. Heatmaps representing the log-transformed ($\log_2$(count + 1)) gene counts across orthogroups (rows) and species (columns) for each deleted block. The color gradient indicates gene counts, where darker colors represent higher gene counts and lighter colors represent lower gene counts. Only orthogroups specific to each block were included.

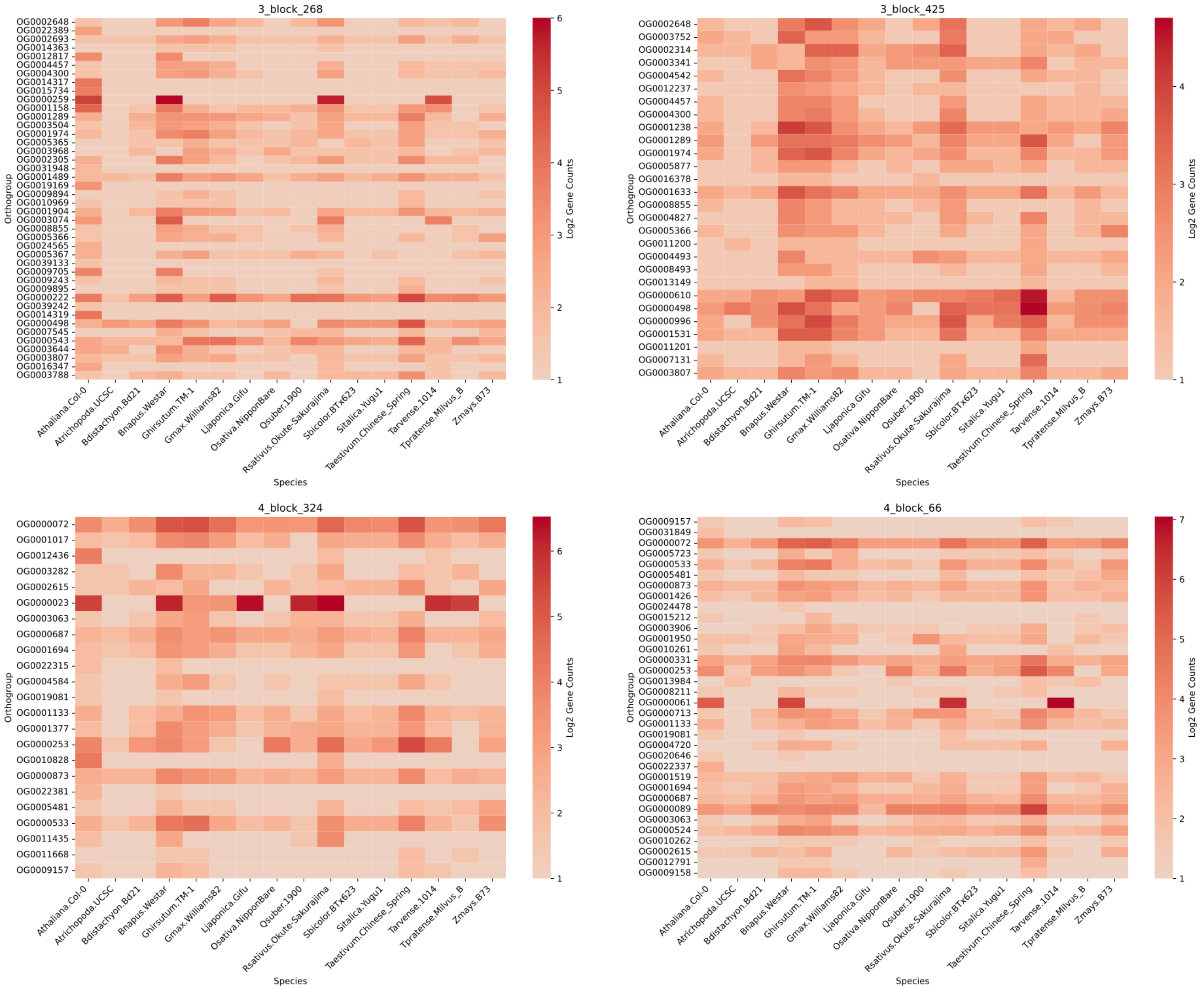

Fig. S2. Heatmaps representing the log-transformed ($\log_2(\text{count} + 1)$) gene counts across orthogroups (rows) and species (columns) for each deleted block (**Fig. S1** continued). The color gradient indicates gene counts, where darker colors represent higher gene counts and lighter colors represent lower gene counts. Only orthogroups specific to each block were included.

Table S1. Frequency of paralogues and single copy genes within each deleted block.

	# of genes with paralogues	frequency of total genes in deleted block	# of single copy genes	frequency of total genes in deleted block
Block 271	25	0.926	2	0.074
Block 438	14	0.875	2	0.125
Block 268	42	0.7	18	0.3
Block 324	31	0.969	1	0.031

The number of genes with paralogues (syntenic paralogues, tandem duplicates, or member of an orthogroup with more than 1 gene) is indicated (1st column) along with the frequency (2nd column) for each deleted block. The number (3rd column) and frequency (4th column) of single copy genes (no identified paralogues) are also provided.

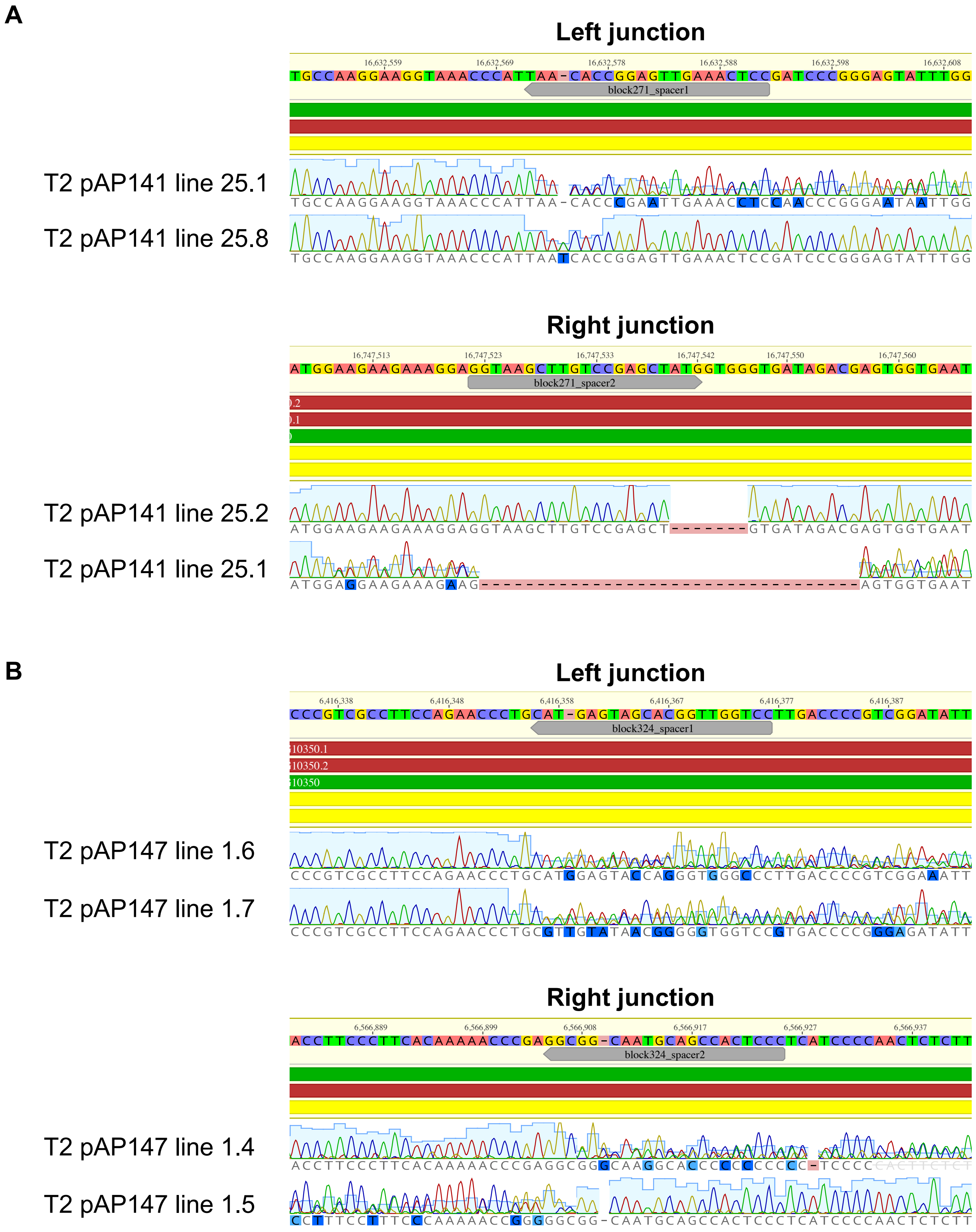

Fig. S3. Verification of gRNA activity at junction sites in plants transformed with pAP141 and pAP147. A) and B) Left and right junction regions of T2 pAP141 and T2 pAP147 plants were amplified and sent for Sanger sequencing to confirm gRNA activity by identifying double strand breaks in transgenic lines. Spacers are annotated within the reference sequence. Bases highlighted in blue represent mismatches.

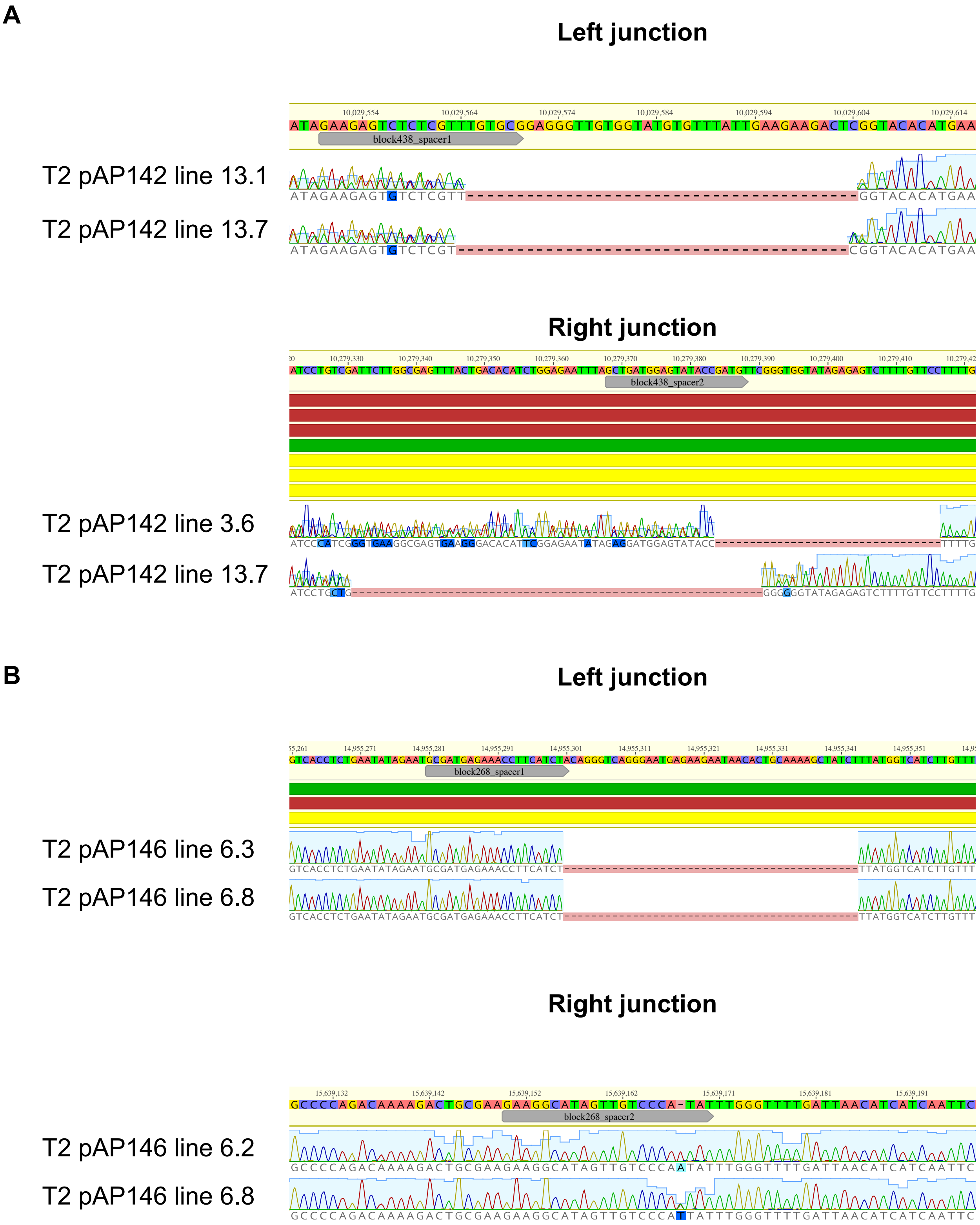

Fig. S4. Verification of gRNA activity at junction sites in plants transformed with pAP142 and pAP146. A) and B) Left and right junction regions of T2 pAP142 and T2 pAP146 plants were amplified and sent for Sanger sequencing to confirm gRNA activity by identifying double strand breaks in transgenic lines. Spacers are annotated within the reference sequence. Bases highlighted in blue represent mismatches.

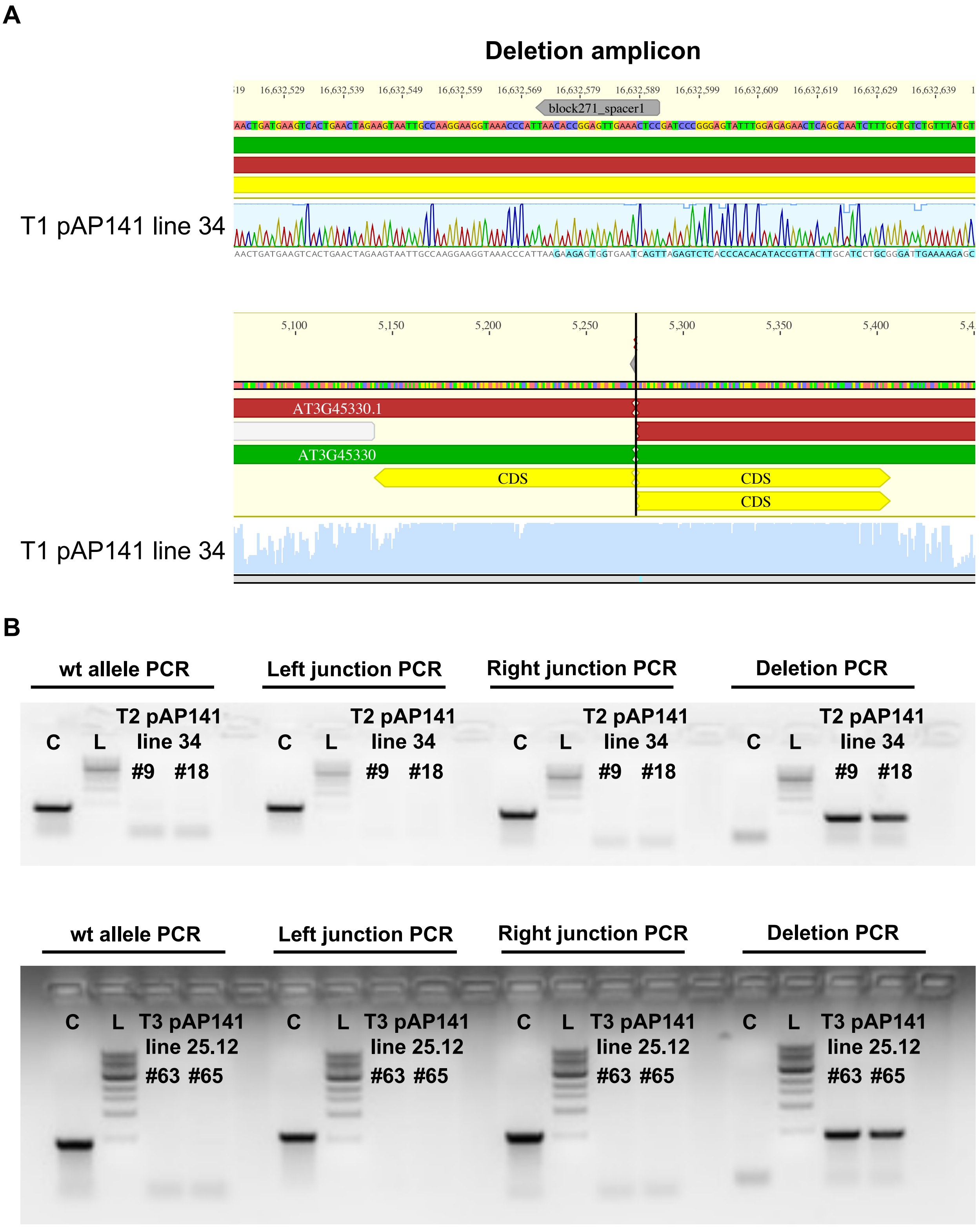

Fig. S5. Deletion of syntenic block 271. A) Top panel displays a screenshot from Geneious of Sanger sequencing results from a deletion amplicon. The spacer sequence is annotated and bases that are highlighted in blue indicate mismatches relative to the reference sequence. Bottom panel represents a zoomed-out view of the top panel with an *in silico* deletion to highlight that the read aligns to the deleted region. The black vertical bar represents the deletion junction. B) wt allele, left junction, right junction, and deletion PCRs were run to identify plants with deletions of both alleles of syntenic block 271. PCRs from T2 and T3 plants from two independent lines are displayed. C=Col-0, L=ladder.

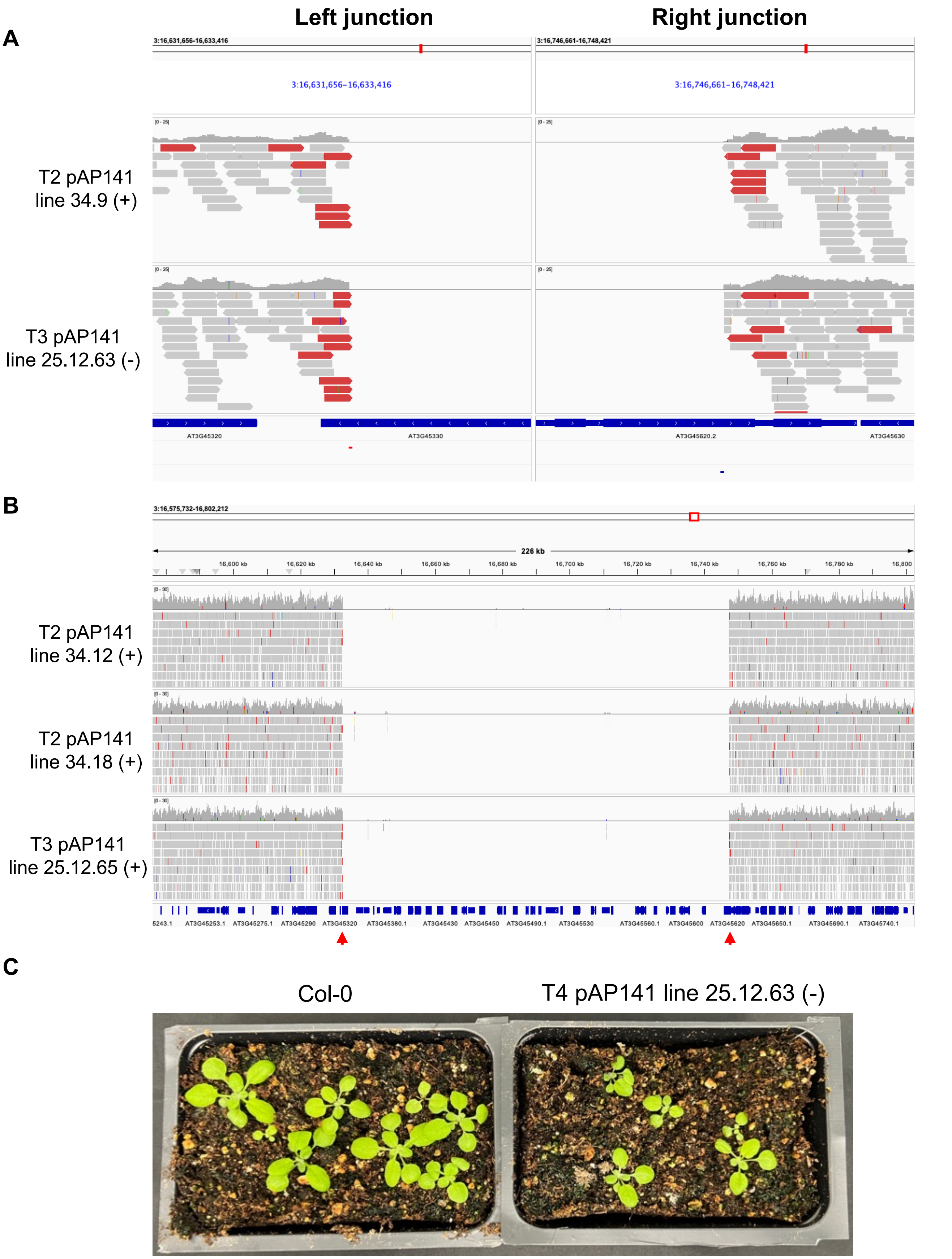

Fig. S6. A) Zoomed in view of Figure 2C displaying left and right junctions of pAP141 samples. Red and blue rectangles beneath alignments represent gRNA sites, where the red rectangle indicates the sequence is from the negative strand and the blue rectangle indicates the sequence is from the positive strand. B) Screenshot from IGV showing whole genome sequencing read alignments within syntenic block 271. Tracks from T2 and T3 samples are displayed. Red arrows represent designed gRNA spacers. C) Comparison of Col-0 and plants from a T4 deletion line. "+" indicates samples containing the T-DNA and "-" indicates null segregants.

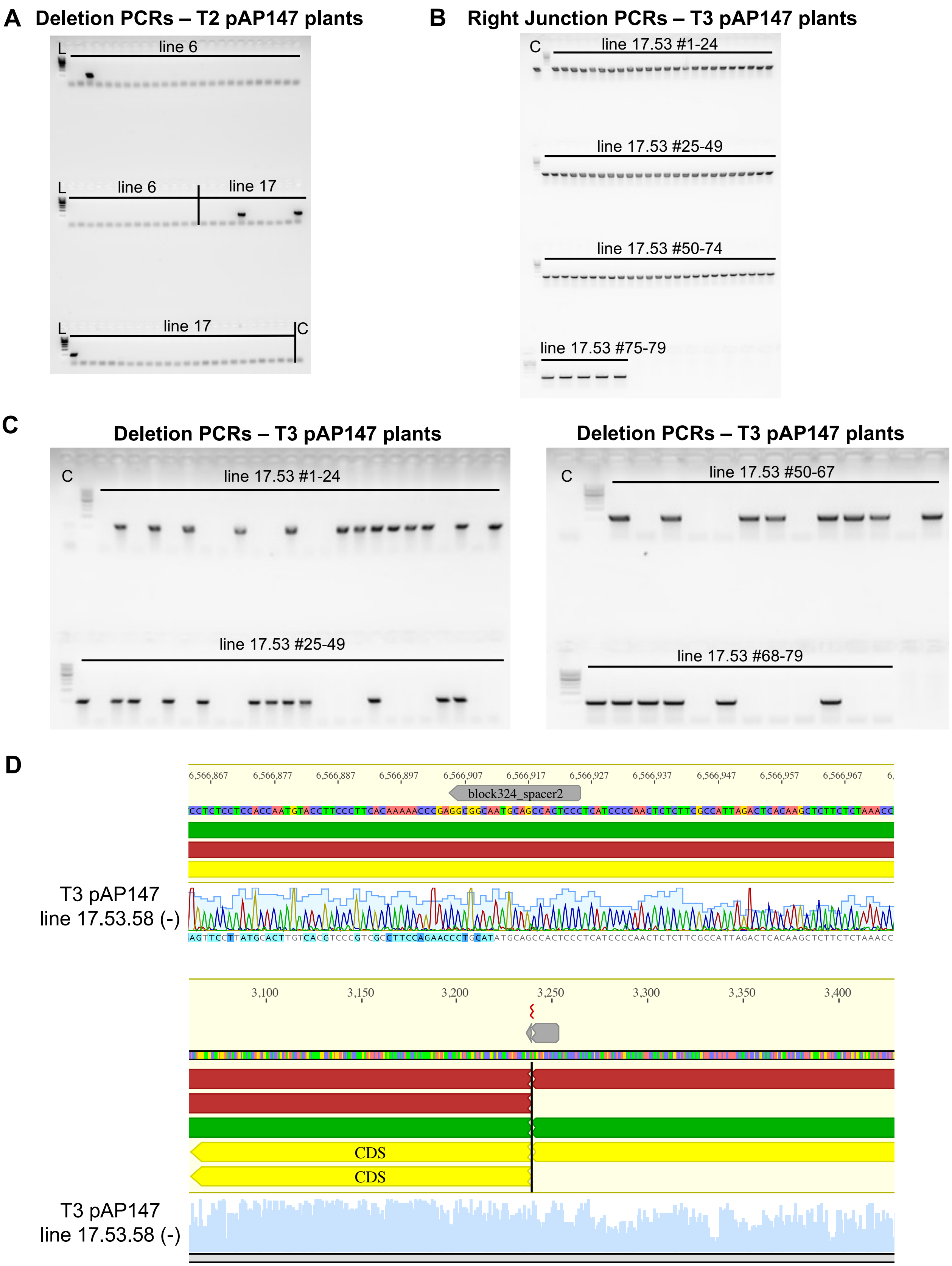

Fig. S7. Deletion of syntenic block 324. A) Deletion PCR screening of individual T2 pAP147 plants from independent lines to identify deletions. C=Col-0, L=ladder. B) Right junction PCRs from a population of T3 plants. C) Both gels display deletion PCR results from T3 plants. D) Top panel displays a screenshot from Geneious of Sanger sequencing results from a deletion amplicon. The spacer sequence is annotated and bases that are highlighted in blue indicate mismatches relative to the reference sequence. Bottom panel represents a zoomed-out view of the top panel with an *in silico* deletion to highlight that the read aligns to the deleted region. The black vertical bar represents the deletion junction."-" indicates samples that are T-DNA null segregants.

A

T3 pAP147
line 1.41.4 (-)

T3 pAP147
line 17.53.52 (-)

B

T3 pAP147
line 1.41.51 (-)

T3 pAP147
line 17.53.58 (-)

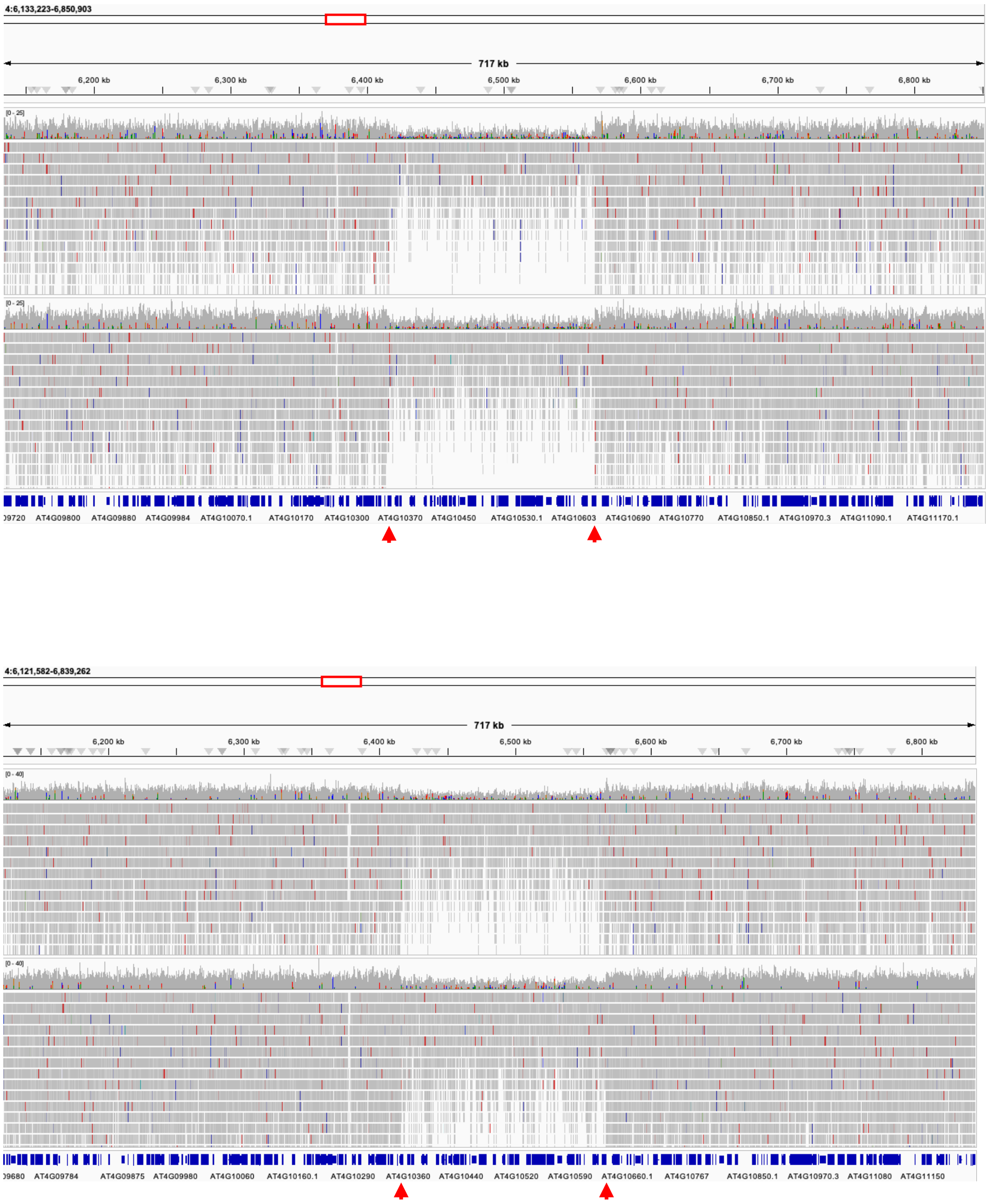

Fig. S8. A-B) Screenshots from IGV showing whole genome sequencing read alignments within syntenic block 324. Tracks from T3 samples from two independent lines and siblings are displayed. Red arrows represent designed gRNA spacers. "-" indicates samples that are T-DNA null segregants.

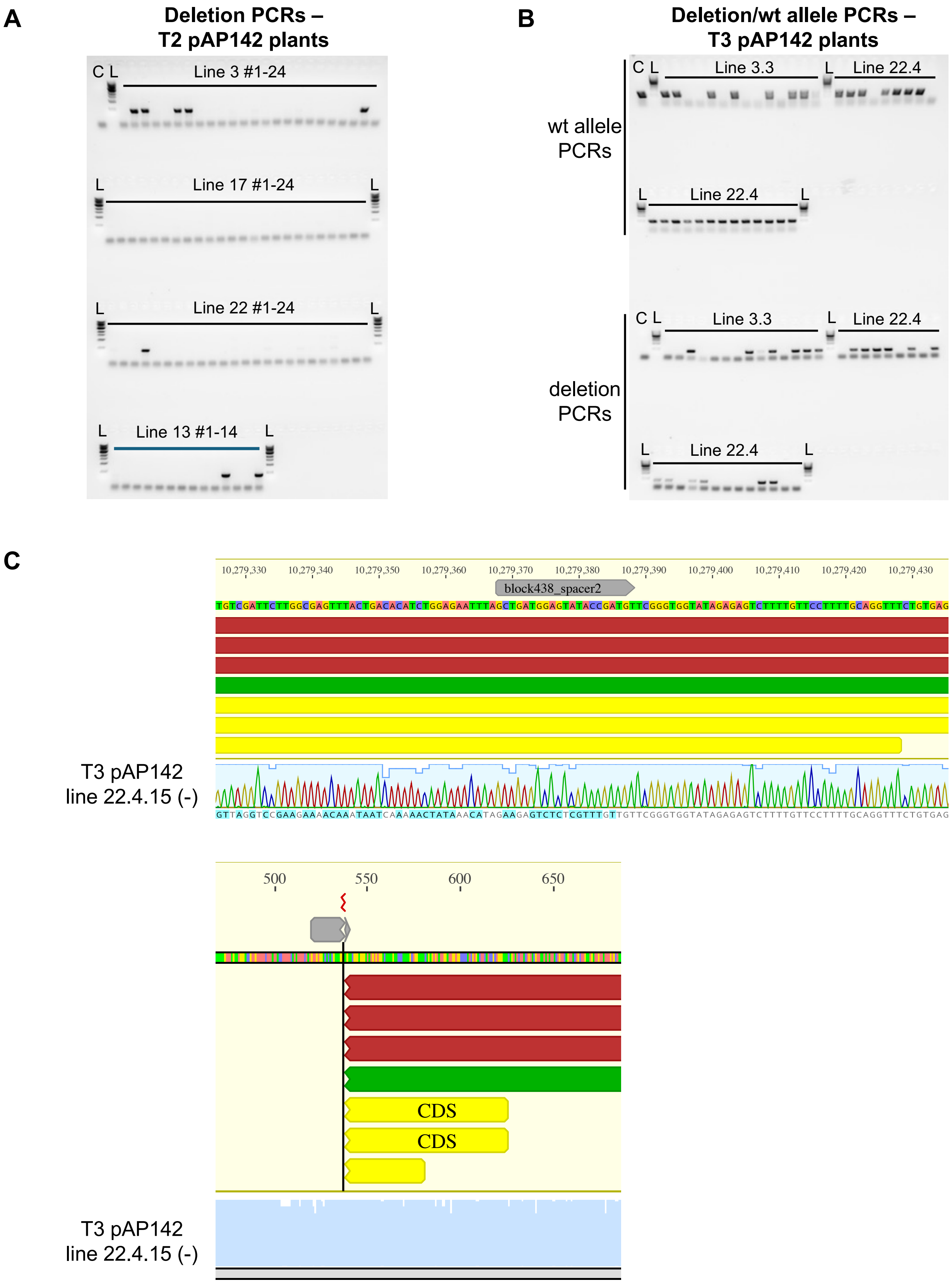

Fig. S9. Deletion of syntenic block 438. A) Deletion PCR screening of individual T2 pAP142 plants from independent lines to identify deletions. C=Col-0, L=ladder. B) Deletion and wt allele PCRs from a population of T3 plants to determine which plants have both alleles of the syntenic block deleted. C) Top panel displays a screenshot from Geneious of Sanger sequencing results from a deletion amplicon. The spacer sequence is annotated and bases that are highlighted in blue indicate mismatches relative to the reference sequence. Bottom panel represents a zoomed-out view of the top panel with an *in silico* deletion to highlight that the read aligns to the deleted region. The black vertical bar represents the deletion junction. "-" indicates samples that are T-DNA null segregants.

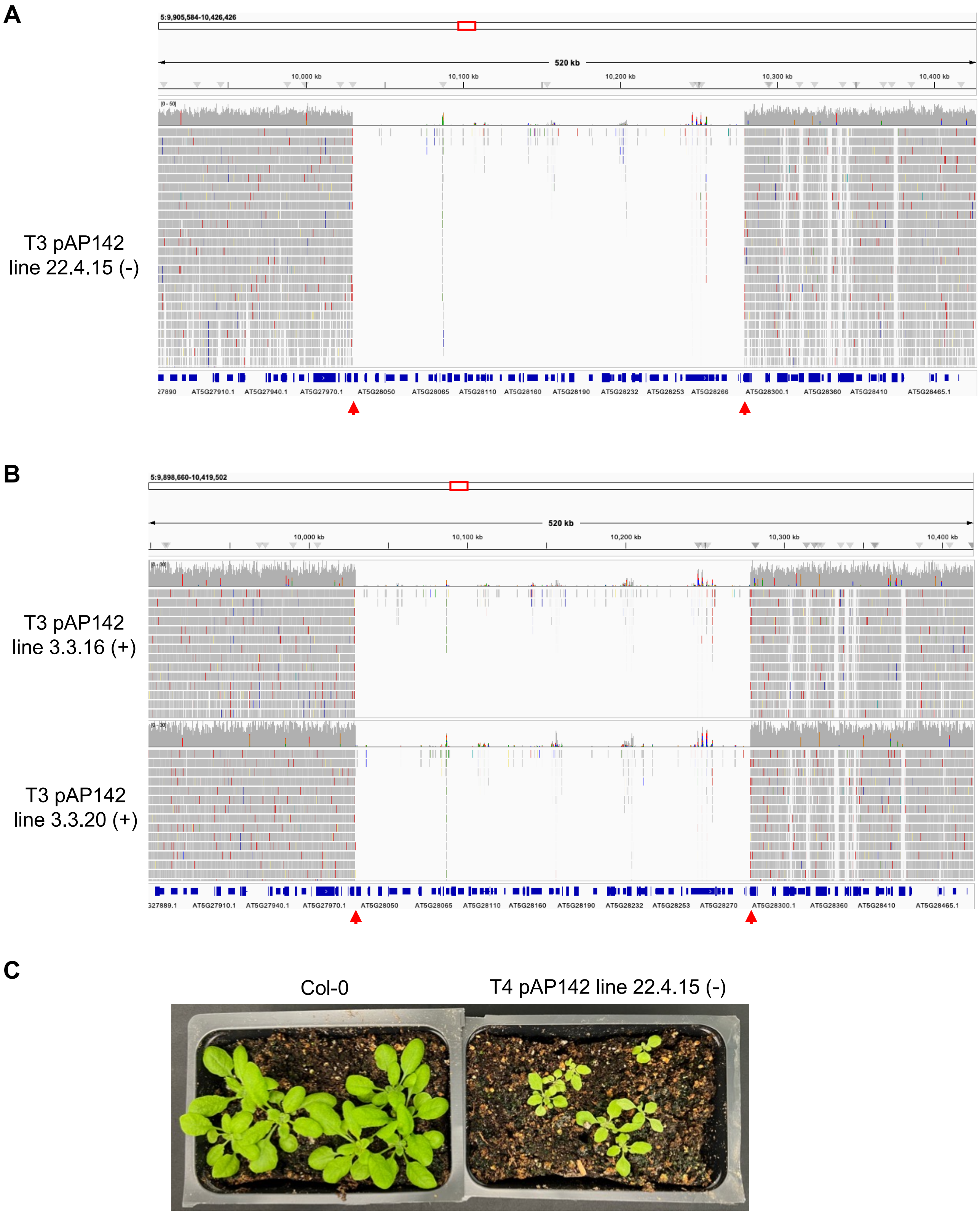

Fig. S10. A-B) Screenshot from IGV showing whole genome sequencing read alignments within syntenic block 438. Tracks from independent T3 lines are displayed. Red arrows represent designed gRNA spacers. C) Comparison of Col-0 and plants from a T4 deletion line. “+” indicates samples containing the T-DNA and “-” indicates null segregants.

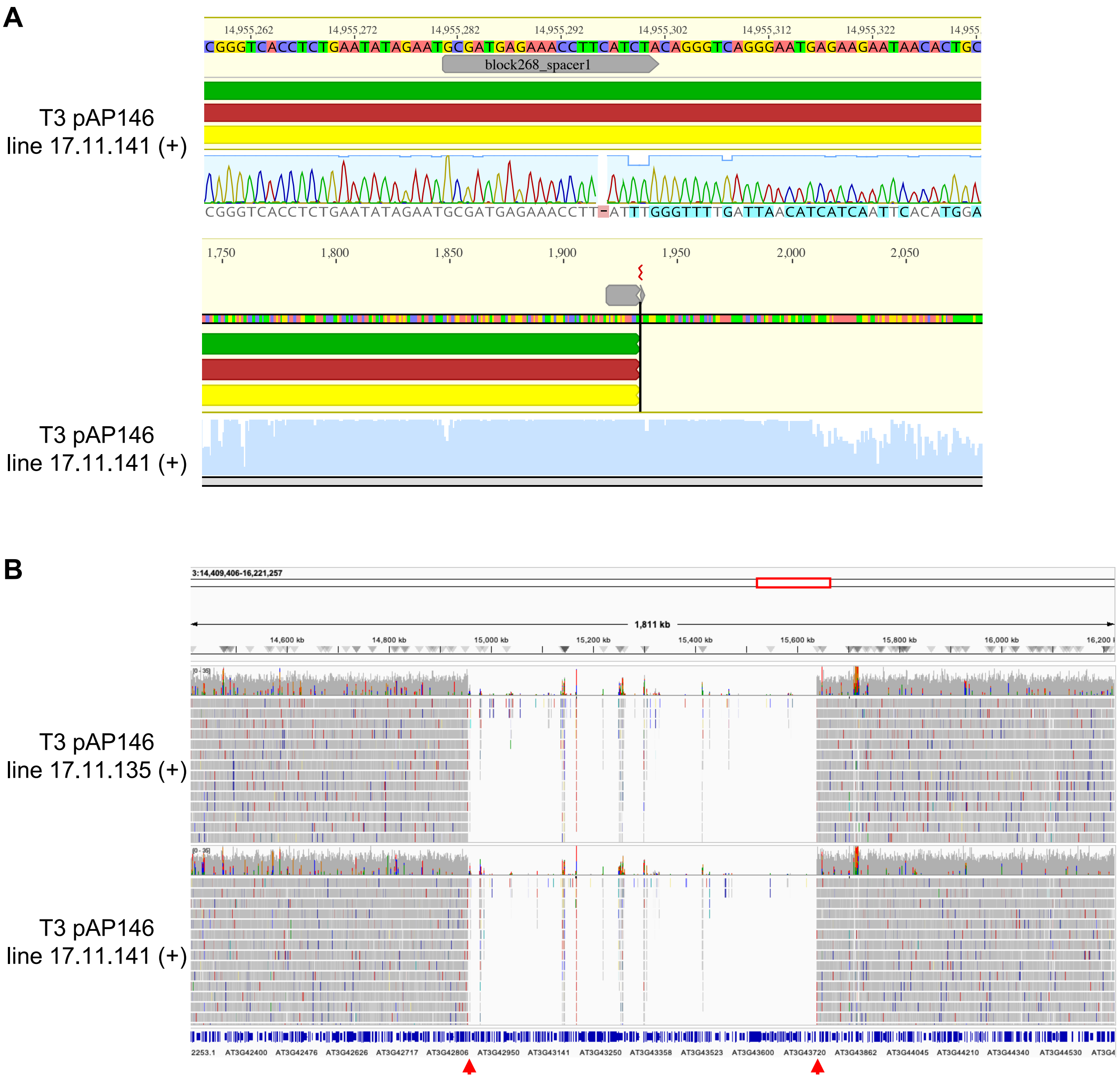

Fig. S11. Deletion of a ~684 kb fragment from block 268. A) The top panel displays a screenshot from Geneious of Sanger sequencing results from deletion amplicon. The spacer sequence is annotated and bases that are highlighted in blue indicate mismatches relative to the reference sequence. The bottom panel represents a zoomed-out view of the top panel with an *in silico* deletion to highlight that the read aligns to the deleted region. The black vertical bar represents the deletion junction. B) Screenshot from IGV showing whole genome sequencing read alignments within syntenic block 268. Tracks from two different plants from a T3 line are displayed. Red arrows represent designed gRNA spacers.

A

T3 pAP146
line 17.11.139 (+)

T3 pAP146
line 17.11.144 (+)

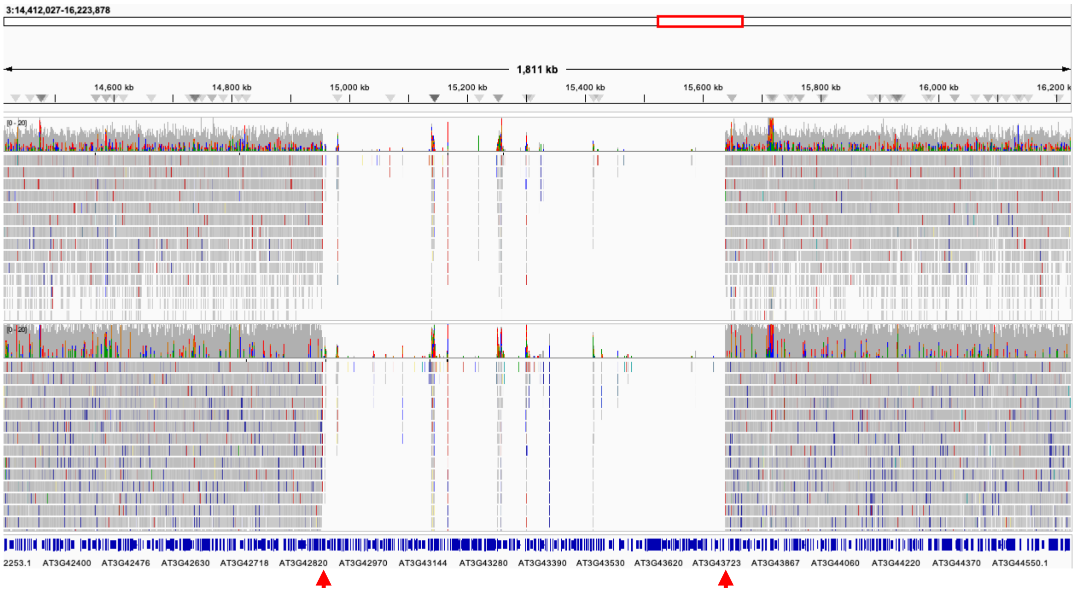

B

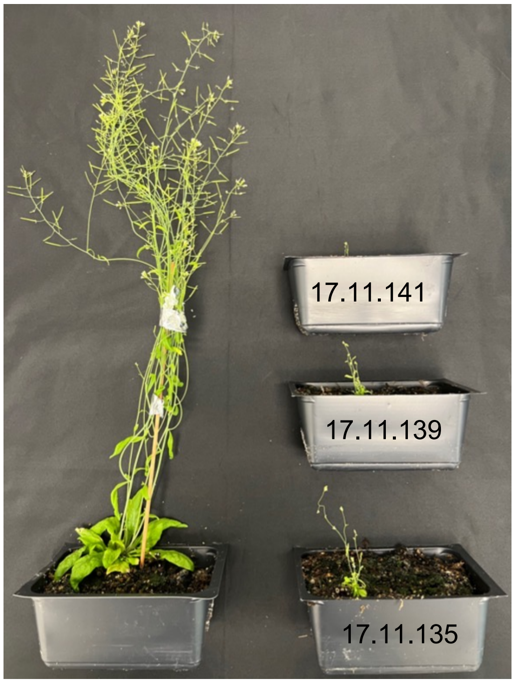

T3 pAP146 line
17.11.126 (het.)

T3 pAP146
deletion lines

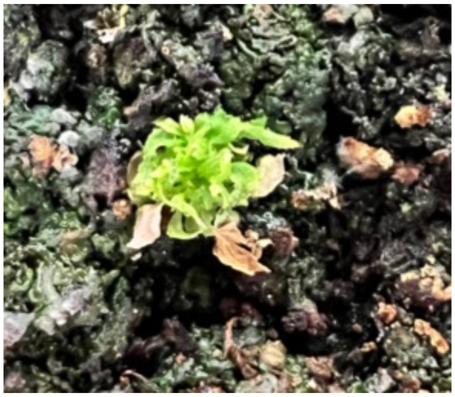

T3 pAP146
line 17.11.170 (+)

Fig. S12. A) Screenshot from IGV showing whole genome sequencing read alignments within syntenic block 268. Tracks from two different plants from a T3 line are displayed. Red arrows represent designed gRNA spacers. B) The first panel depicts a plant heterozygous for the deletion (het.) compared to 3 different T3 plants that are homozygous. The second panel shows a close-up view of a block 268 deletion line. “+” indicates samples that contain the T-DNA.

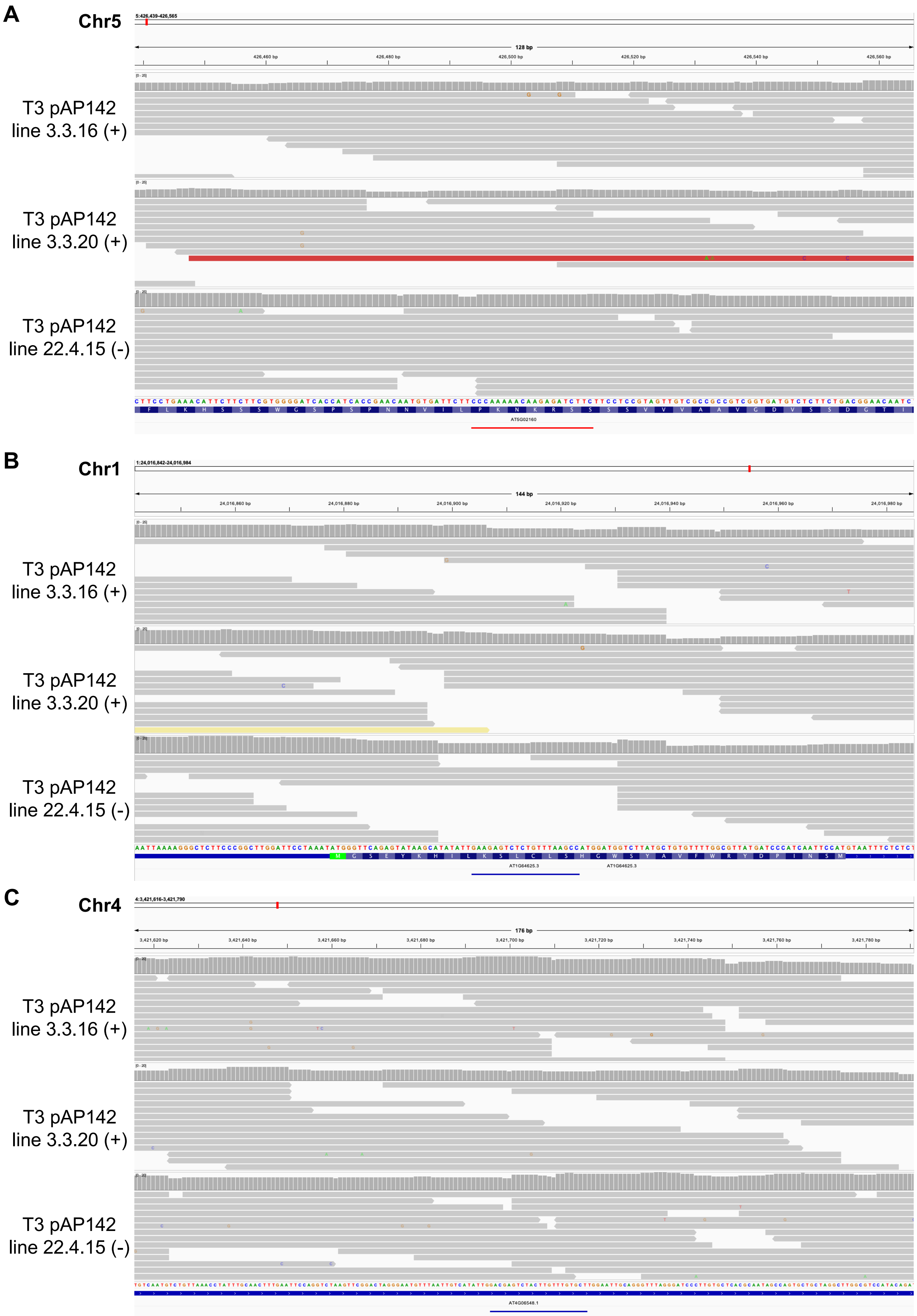

Fig. S13. A-C) Screenshot from IGV showing whole genome sequencing read alignments at the three predicted off-target sites (from Cas-OFFinder) for gRNA1 from pAP142. Tracks from three different plants from T3 lines are displayed. Red (minus strand) or blue (plus strand) bars beneath tracks represent predicted off target binding sites.

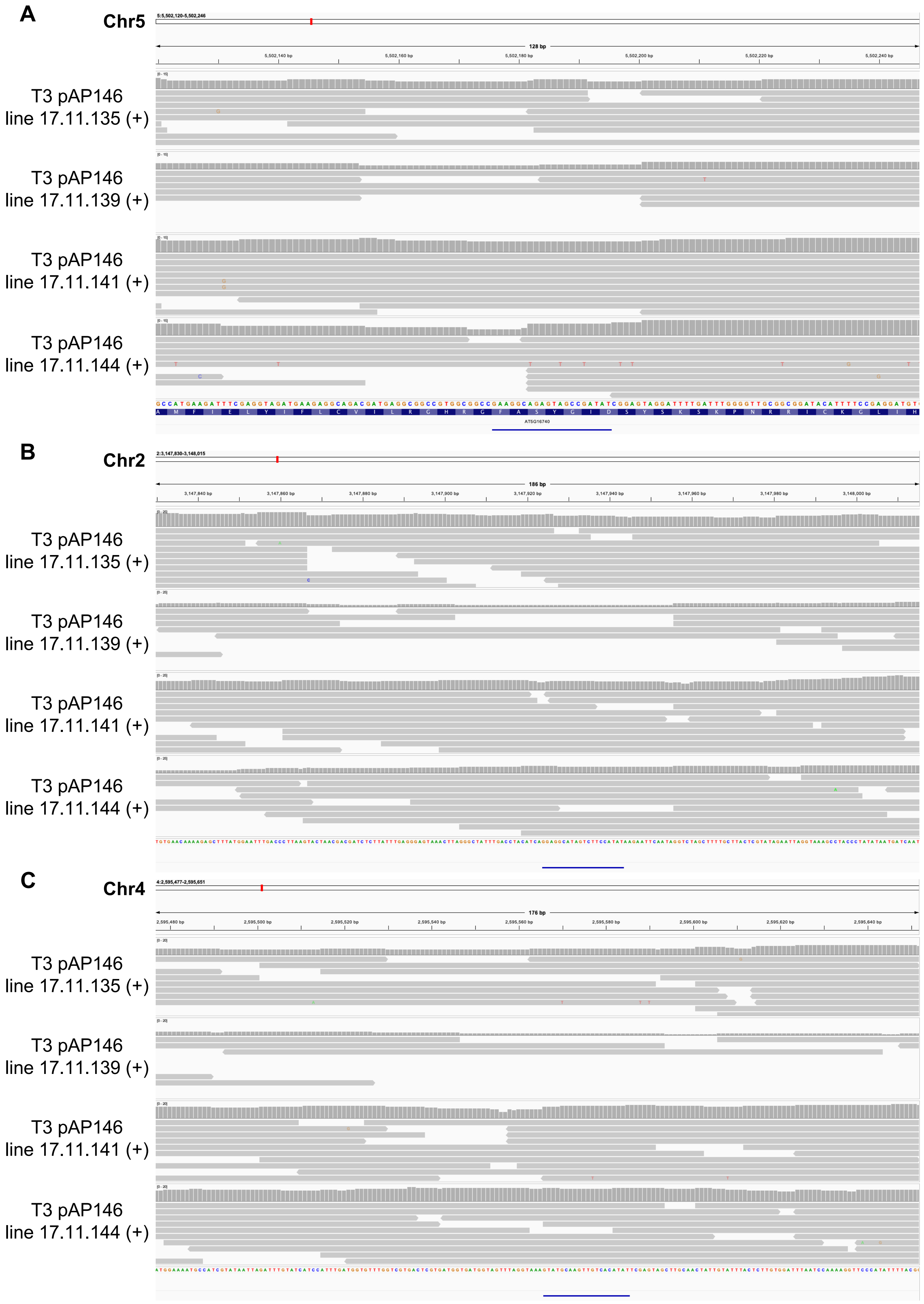

Fig. S14. A-C) Screenshot from IGV showing whole genome sequencing read alignments at the three predicted off-target sites (from Cas-OFFinder) for gRNA2 from pAP146. Tracks from four different plants from a T3 line are displayed. Blue bars beneath tracks represent predicted off target binding sites.

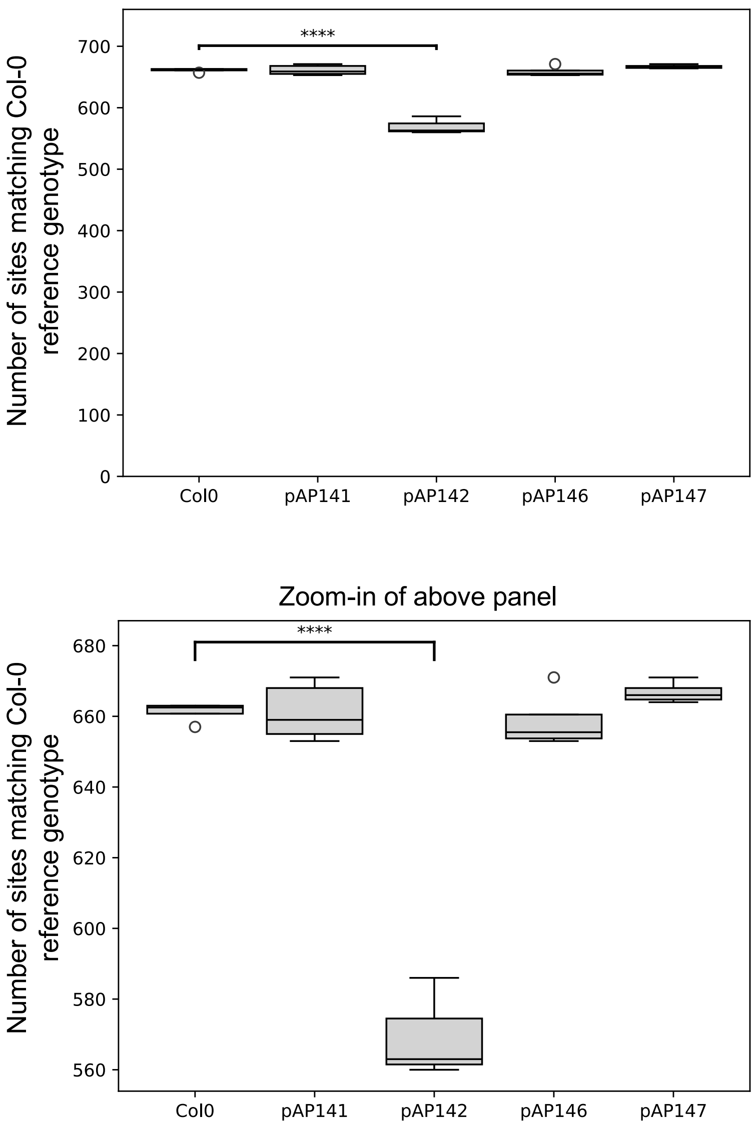

Fig. S15. Box plots displaying off-target effects of syntenic block editing. The y-axis represents the total number of sites matching the Col-0 reference genome (homozygous). n=4 for Col-0, n=5 for pAP141, n=3 for pAP142, n=4 for pAP146, n=4 for pAP147. Dunnett's test reveals that the difference between Col-0 vs. pAP142 is statistically significant (p=6.14E-13). Bottom panel represents a zoomed in version of the top panel.

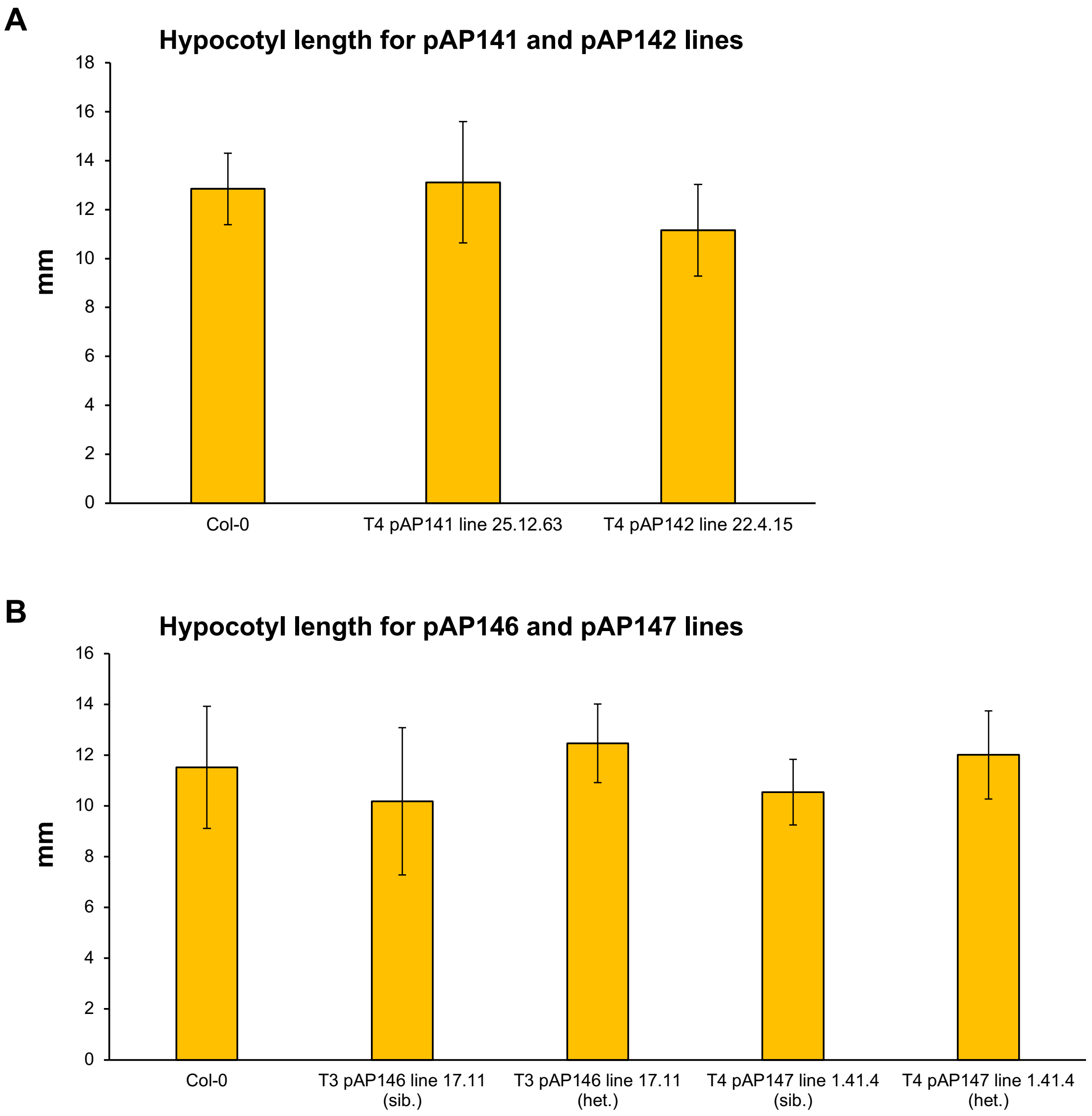

Fig. S16. Hypocotyl length measurements of deletion lines. A) Average hypocotyl lengths (in millimeters) of Col-0, pAP141, and pAP142 lines. n=11 for Col-0, n=12 for pAP141, and n=12 for pAP142. B) Average hypocotyl lengths of Col-0, wt sibling controls, and heterozygous pAP146 and pAP147 lines. n=8 for Col-0, n=4 for pAP146 (sib.), n=9 for pAP146 (het.), n=3 for pAP147 (sib.), and n=9 for pAP147 (het.). Error bars represent standard deviation. sib. = wt sibling control. het. = heterozygous for the deletion.

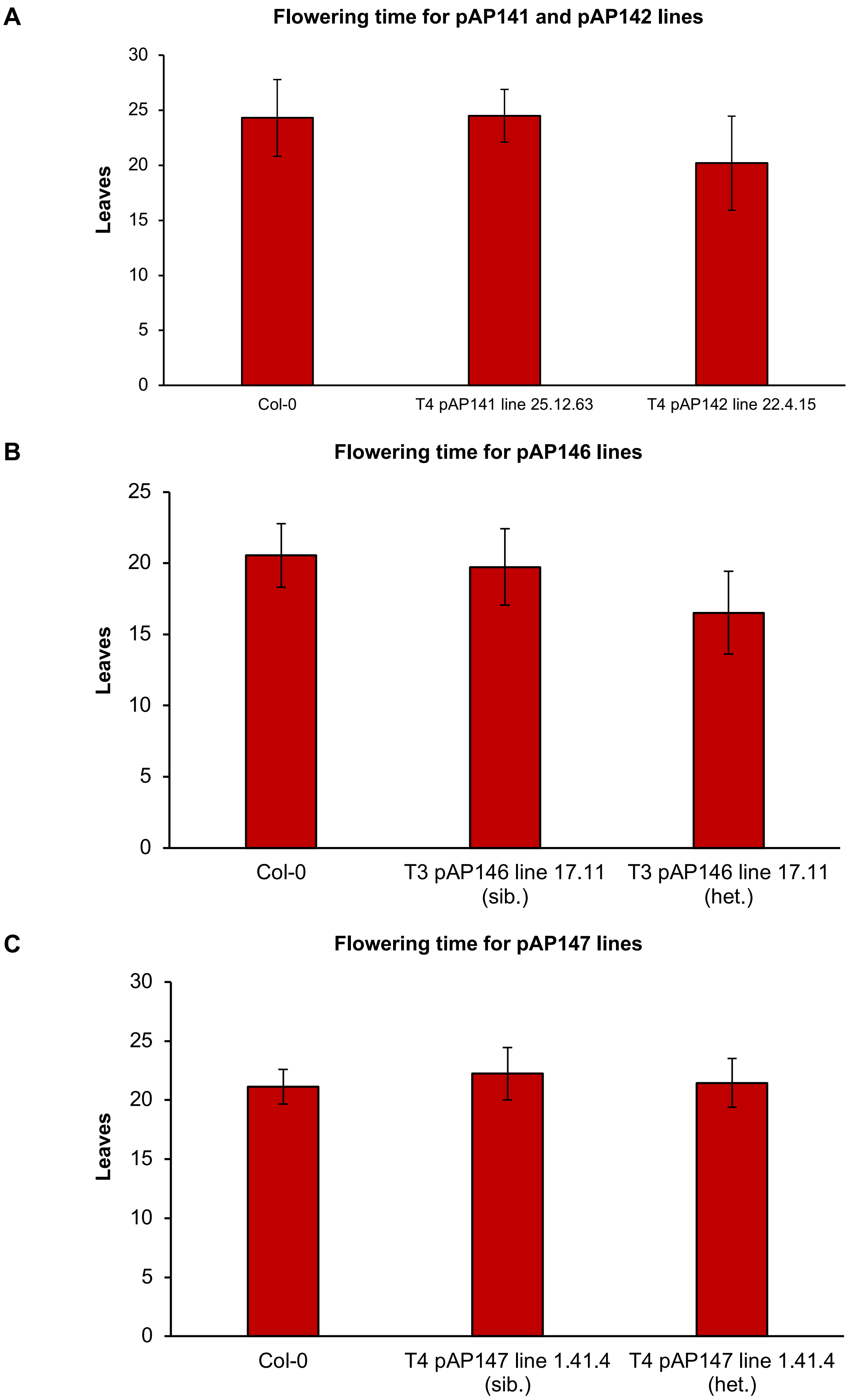

Fig. S17. Deletion line flowering time assays. A) Average number of leaves during flowering for Col-0, pAP141, and pAP142 lines. n=16 for Col-0, n=12 for pAP141, and n=10 for pAP142. B) Average number of leaves during flowering for Col-0, pAP146 (sib.), and pAP146 (het.). n=22 for Col-0, n=29 for pAP146 (sib.), and n=29 for pAP146 (het.) C) Average number of leaves during flowering for Col-0, pAP147 (sib.), and pAP147 (het.). n=8 for Col-0, n=4 for pAP147 (sib.), and n=11 for pAP147 (het.). Error bars represent standard deviation. sib. = wt sibling control. het. = heterozygous for the deletion.

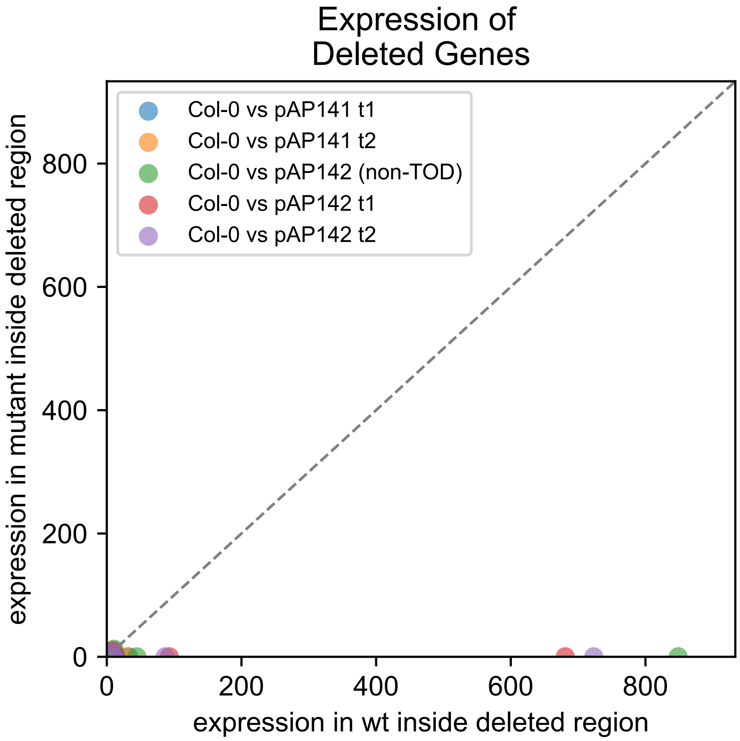

Fig. S18. Investigation of compensation for deleted genes at the transcriptional level. All abundance estimates are in transcripts per million. The plot features a line at Y=X indicating no change in expression in the specified comparison. In Col-0 and the pAP141 and pAP142 deletion lines, total expression of the deleted gene members of orthogroups shows no expression in deletion lines, as expected. t1 = time point 1, t2 = time point 2. non-TOD refers to the separate set of pAP142 samples used for RNA-seq that were not part of the time of day (TOD) experiment.

A

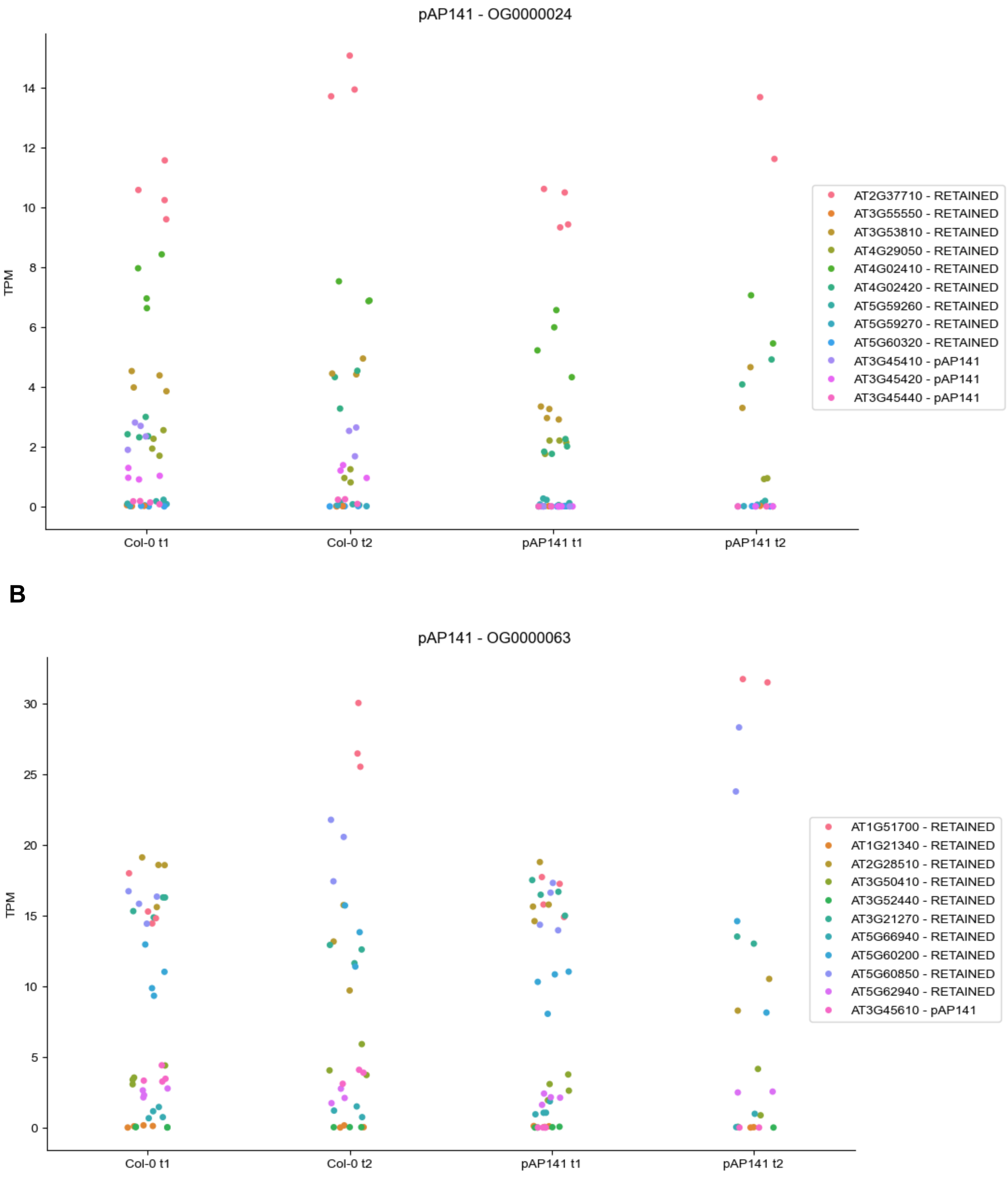

Fig. S19. A-B) Expression of gene members of an orthogroup in pAP141 deletion lines across two timepoints. Expression is indicated as TPM. Jitter has been added to the categorical “sample” axis for visualization purposes. Genes that weren’t deleted are marked as “retained” in the figure. Genes that were deleted are marked with the line name (i.e., pAP141). t1 = time point 1, t2 = time point 2.

A

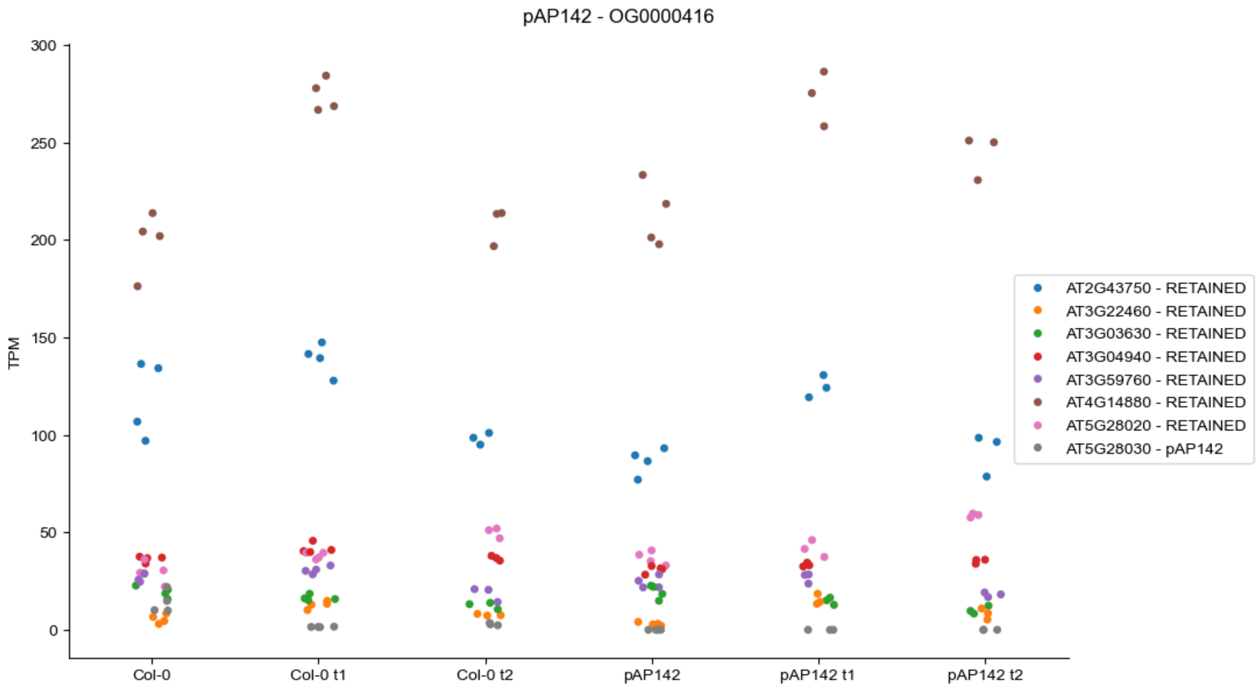

B

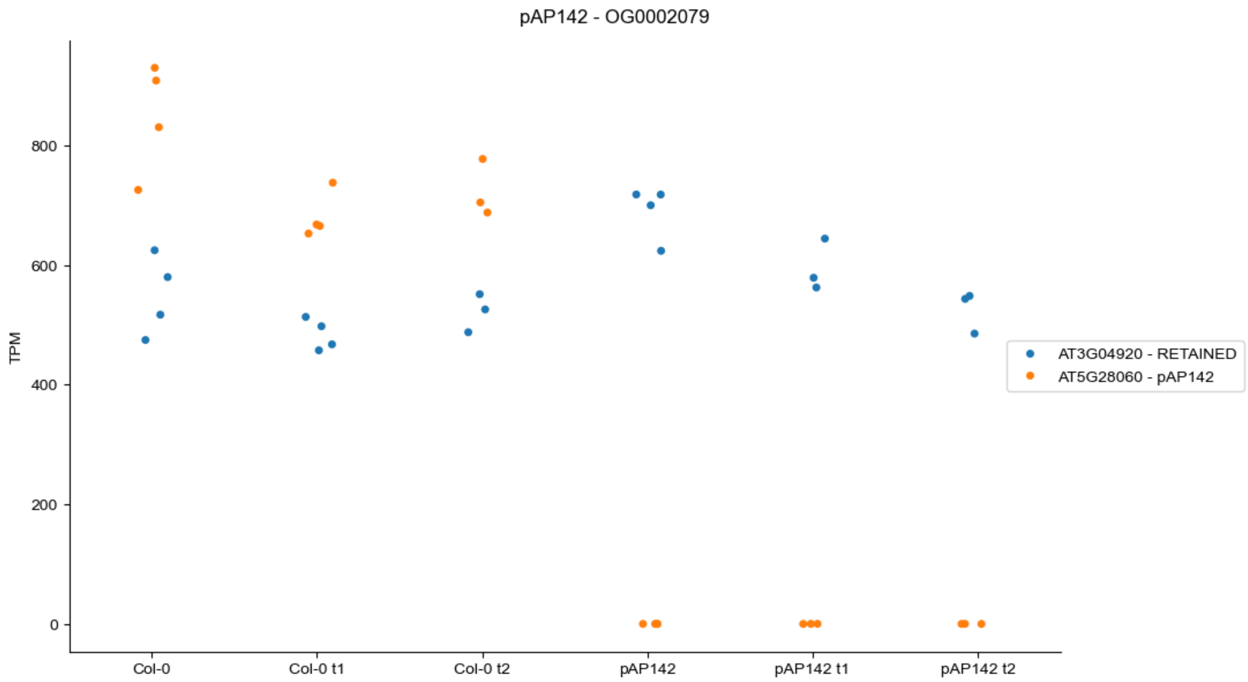

Fig. S20. A-B) Expression of gene members of an orthogroup in pAP142 deletion lines across two timepoints and an additional set of non-TOD pAP142 samples. Expression is indicated as TPM. Jitter has been added to the categorical "sample" axis for visualization purposes. Genes that weren't deleted are marked as "retained" in the figure. Genes that were deleted are marked with the line name (i.e., pAP142). t1 = time point 1, t2 = time point 2.

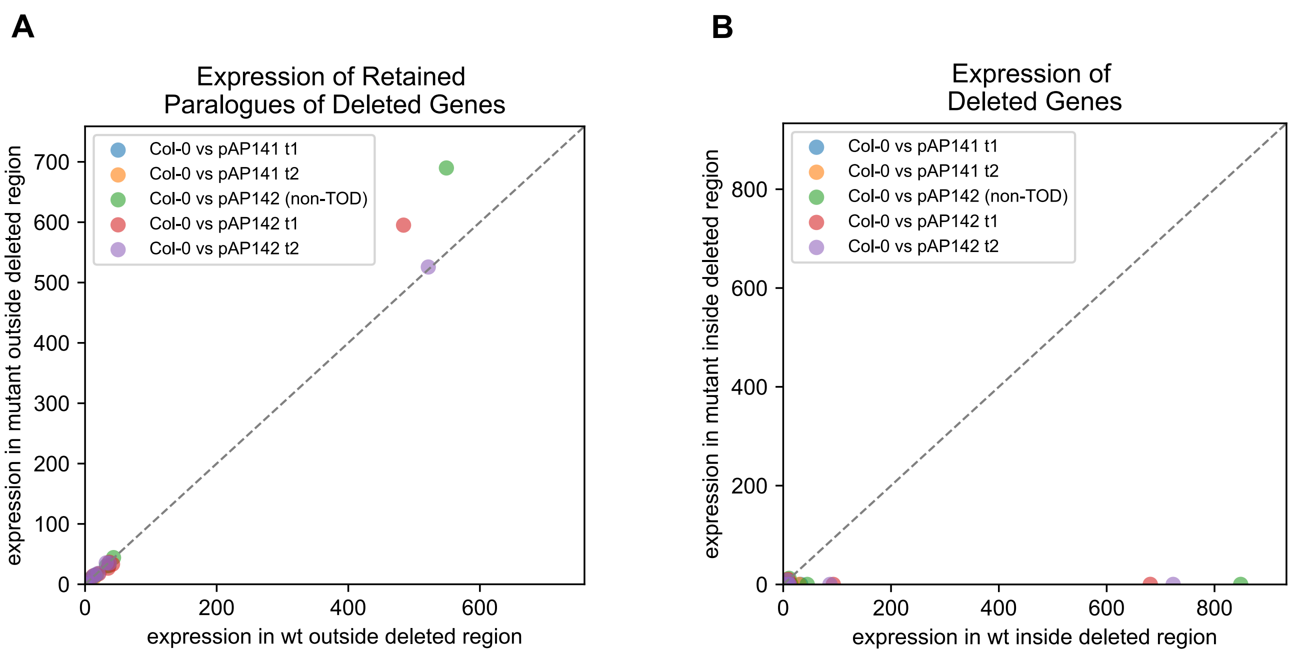

Fig. S21. Investigation of compensation for deleted genes from retained syntenic paralogues in pAP141 and pAP142 lines. All abundance estimates are in transcripts per million. Plots A and B feature a line at Y=X indicating no change in expression in the specified comparison. A) In Col-0 and the pAP141 and pAP142 deletion lines, total expression of the retained syntenic paralogues of deleted genes shows no general upregulation. B) In Col-0 and the pAP141 and pAP142 deletion lines, there is no expression of the deleted genes in deletion lines, as expected. t1 = time point 1, t2 = time point 2. non-TOD refers to the separate set of pAP142 samples used for RNA-seq that were not part of the time of day (TOD) experiment.

A

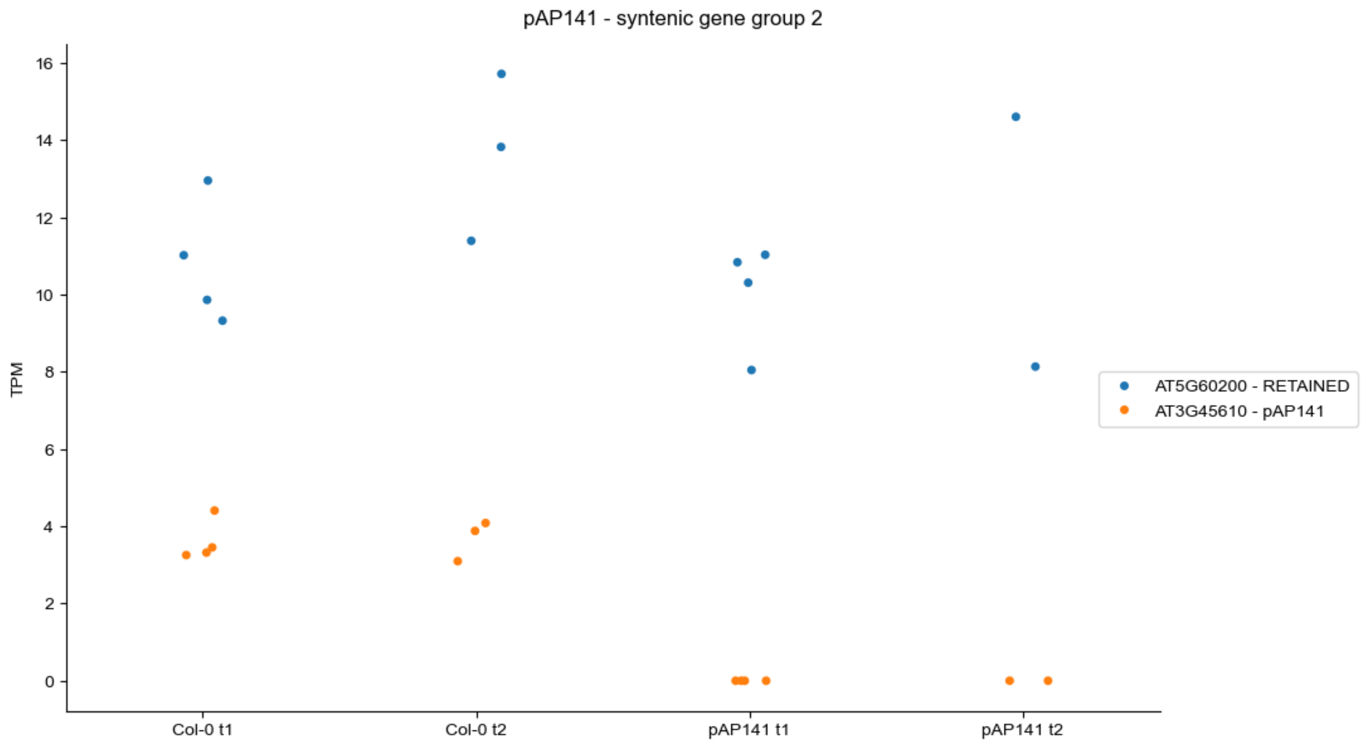

B

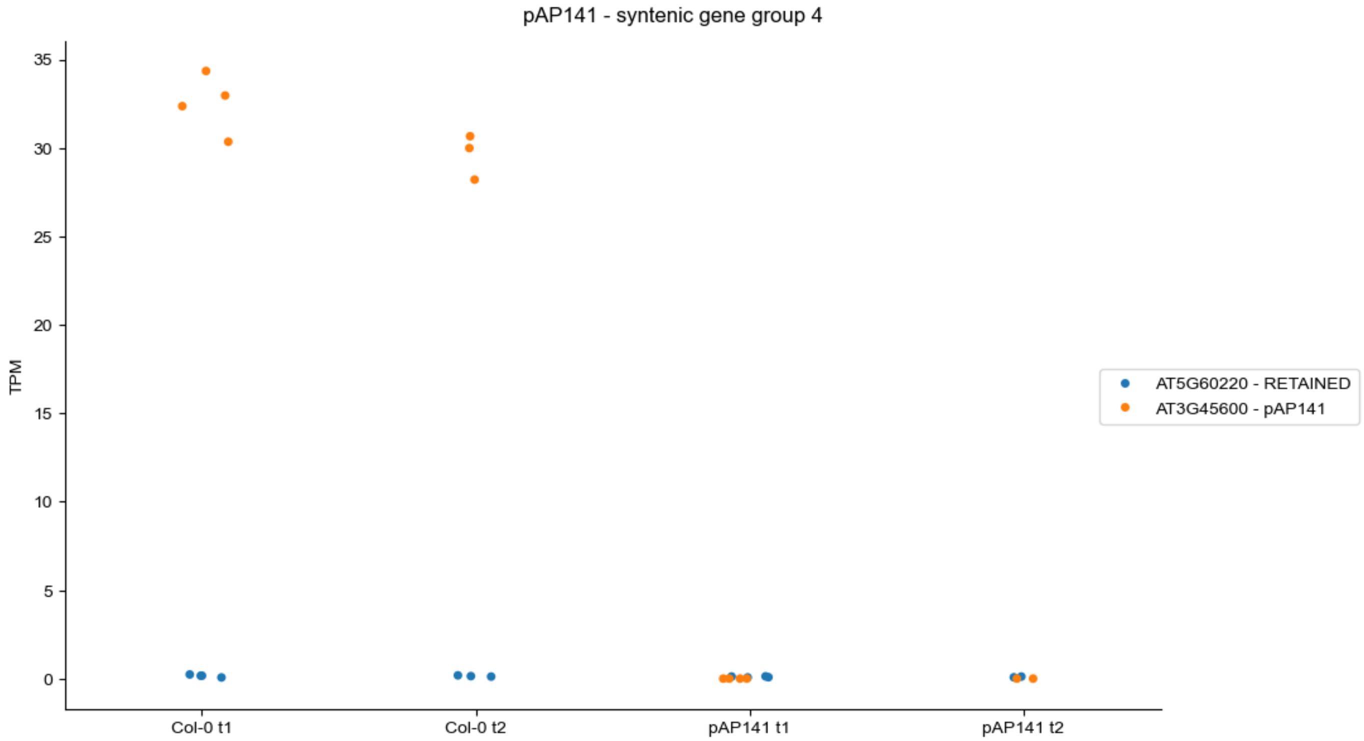

Fig. S22. A-B) Expression of syntenic paralogues in pAP141 deletion lines across two timepoints. Expression is indicated as TPM. Jitter has been added to the categorical “sample” axis for visualization purposes. Genes that weren’t deleted are marked as “retained” in the figure. Genes that were deleted are marked with the line name (i.e., pAP141). t1 = time point 1, t2 = time point 2.

A

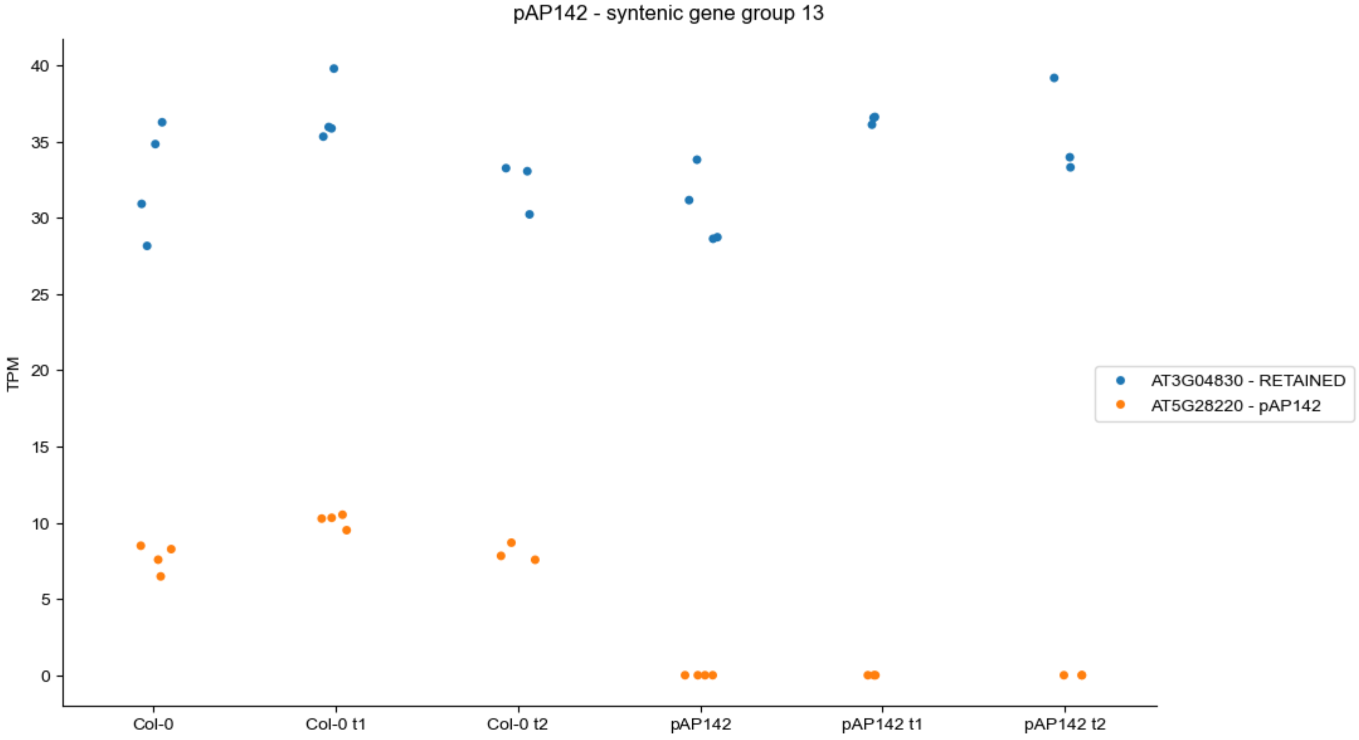

B

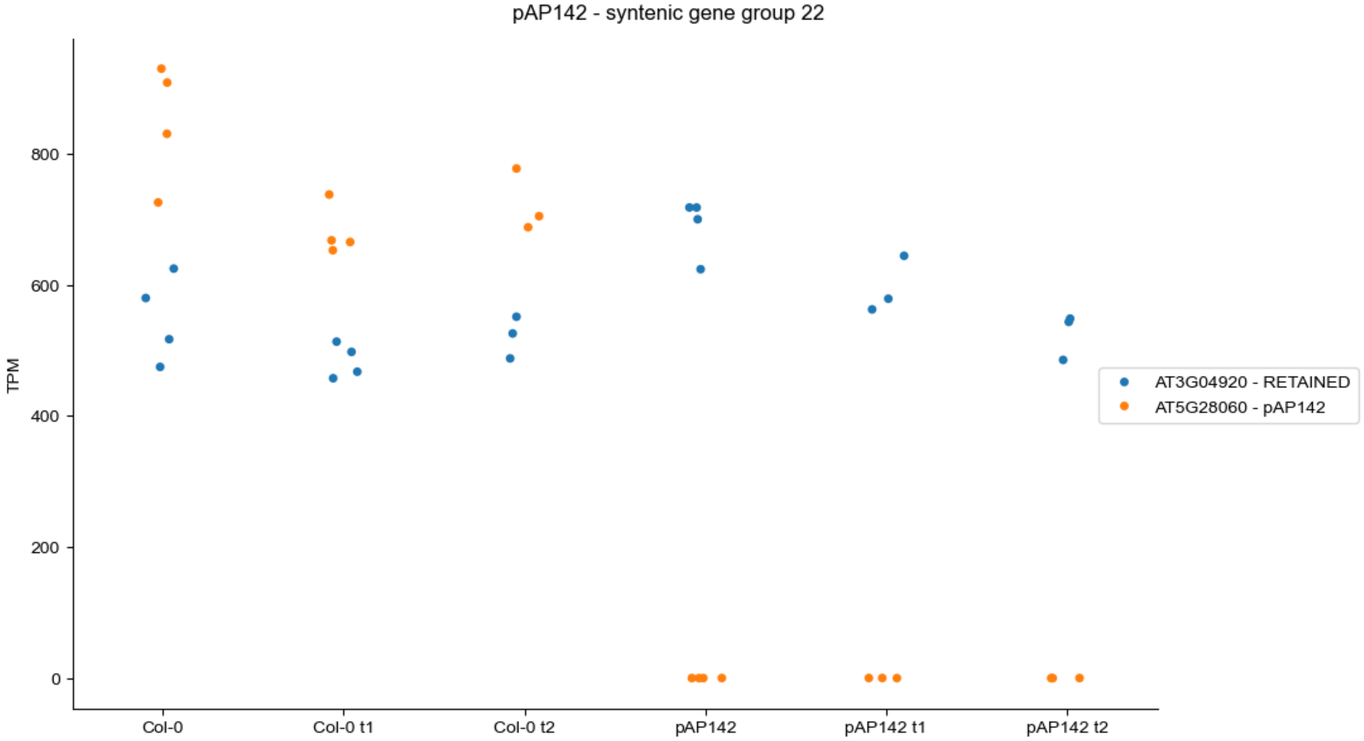

Fig. S23. A-B) Expression of syntenic paralogues in pAP142 deletion lines across two timepoints and an additional set of non-TOD pAP142 samples. Expression is indicated as TPM. Jitter has been added to the categorical “sample” axis for visualization purposes. Genes that weren’t deleted are marked as “retained” in the figure. Genes that were deleted are marked with the line name (i.e., pAP142). t1 = time point 1, t2 = time point 2.

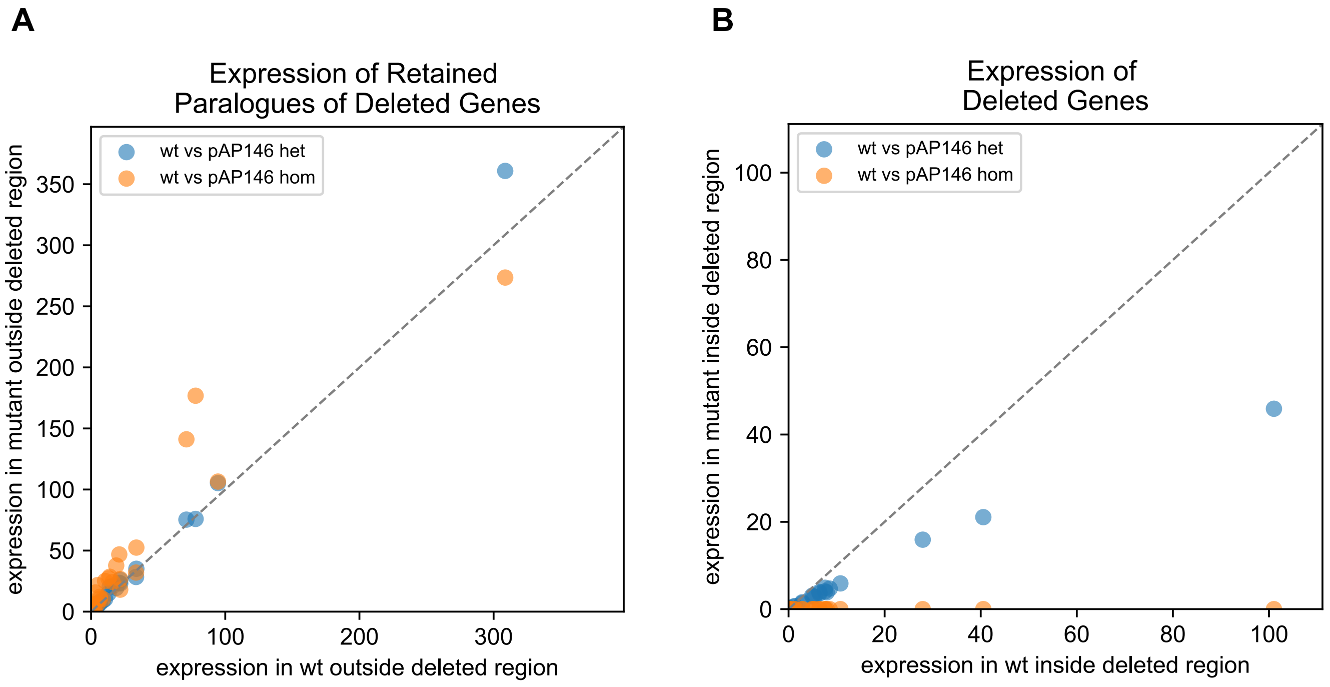

Fig. S24. Investigation of compensation for deleted genes at the transcriptional level. All abundance estimates are in transcripts per million. Plots A and B feature a line at Y=X indicating no change in expression in the specified comparison. A) Total expression of the retained gene members of orthogroups affected by deletions in wt and pAP146 heterozygous and homozygous deletion lines. B) Total expression of the deleted gene members of orthogroups shows no expression in the homozygous deletion lines and approximately 50% of wt expression levels in the heterozygous deletion lines. wt = wild type, which in plots A and B refer to Col-0 and sibling controls. het = heterozygous, hom = homozygous.

A

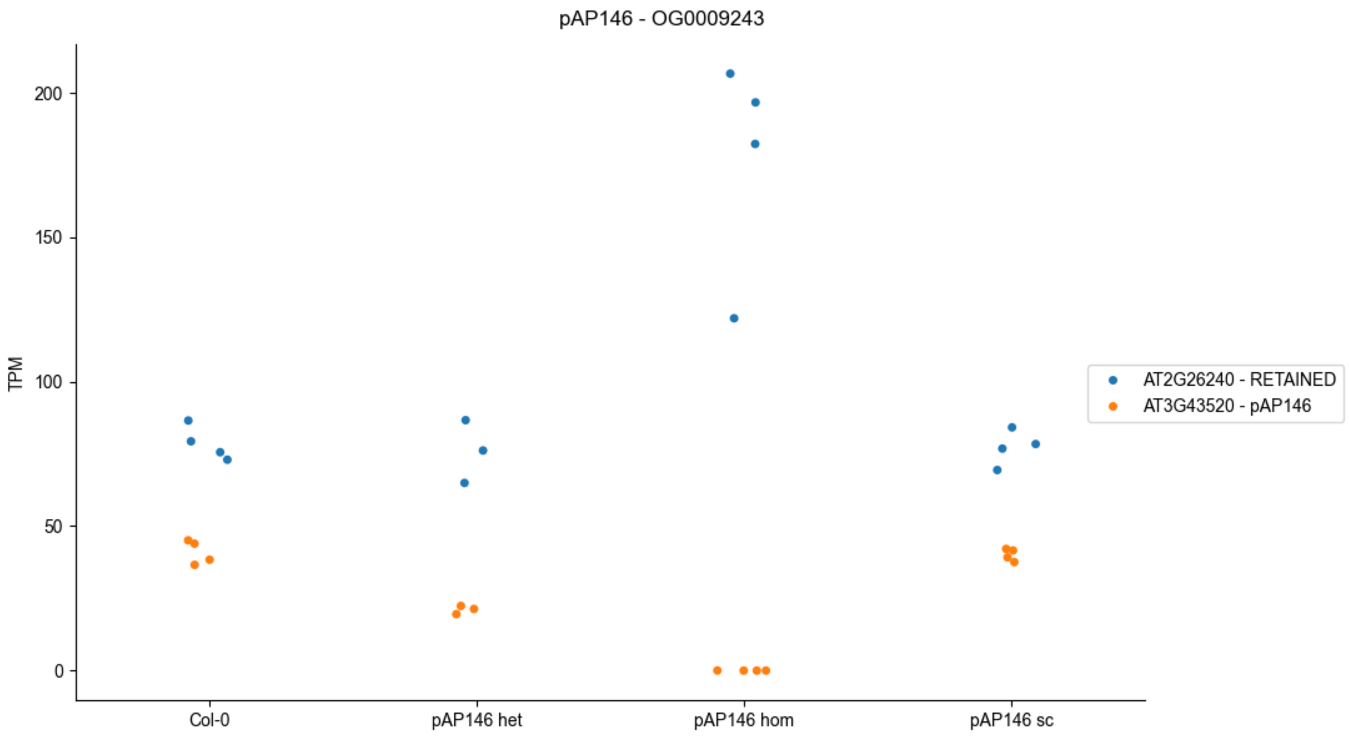

B

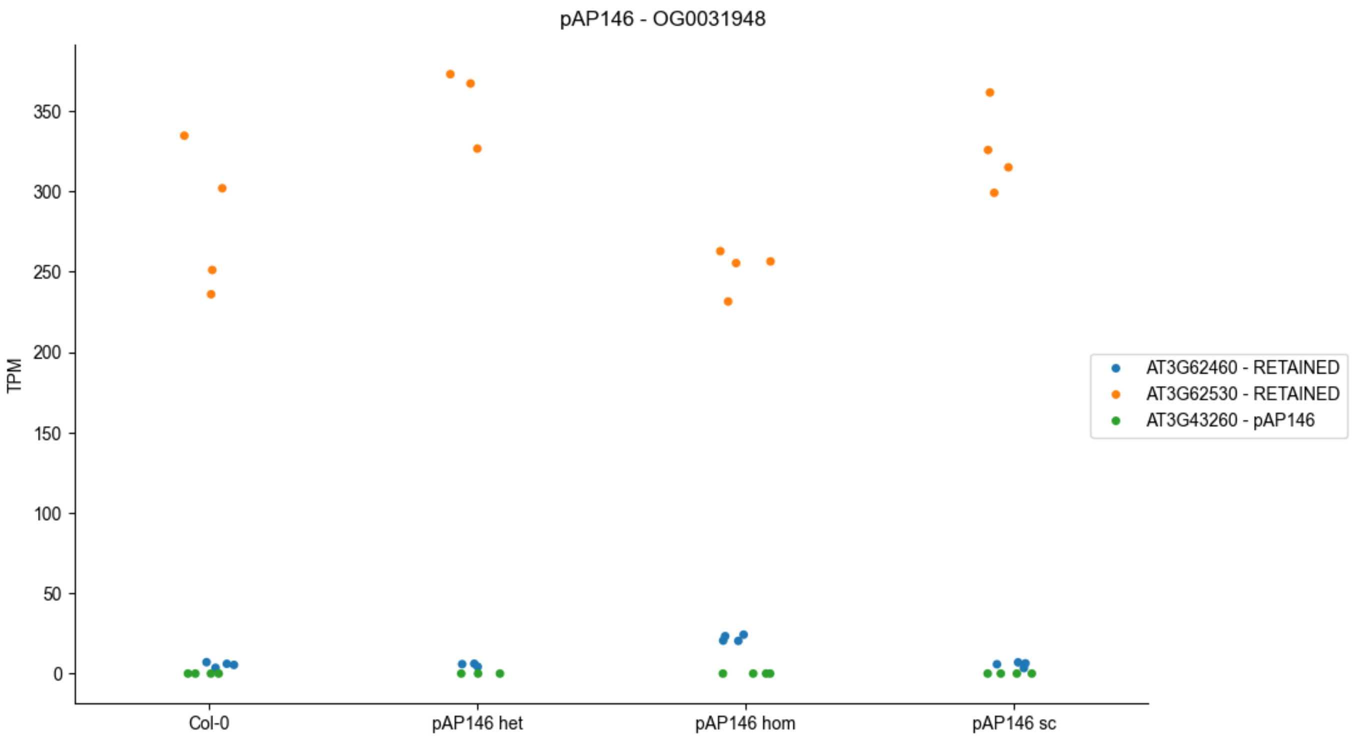

Fig. S25. A-B) Expression of gene members of an orthogroup in pAP146 deletion lines. Expression is indicated as TPM. Jitter has been added to the categorical "sample" axis for visualization purposes. Genes that weren't deleted are marked as "retained" in the figure. Genes that were deleted are marked with the line name (i.e., pAP146). het = heterozygous, hom = homozygous, sc = sibling wt control.

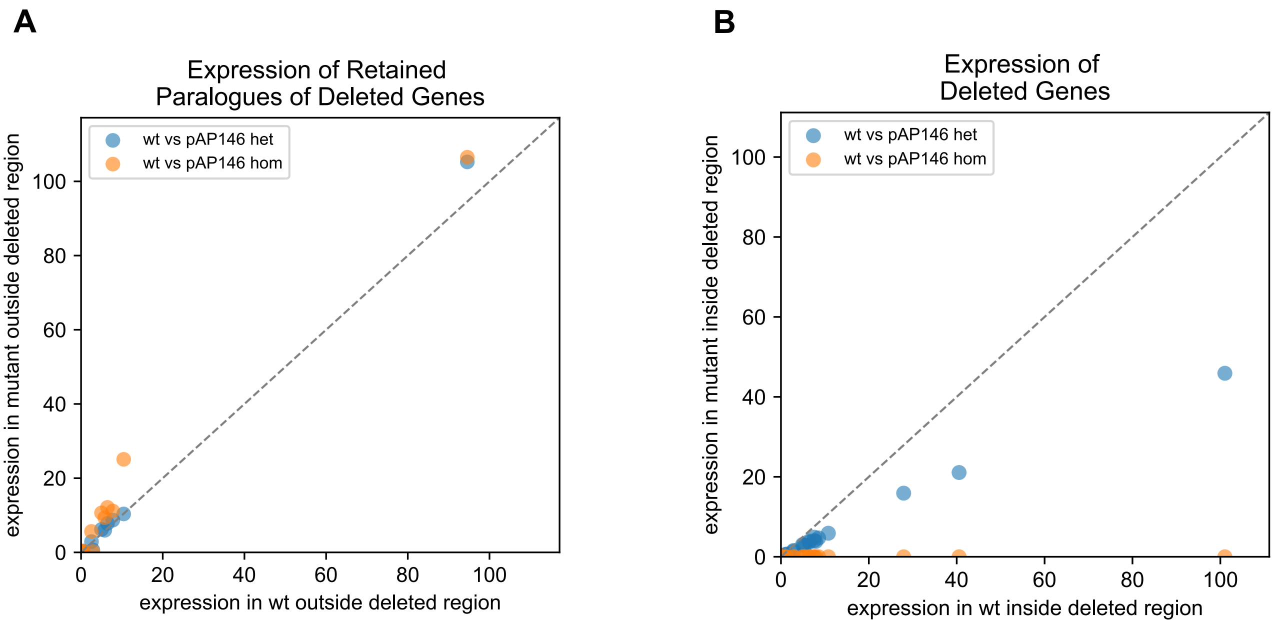

Fig. S26. Investigation of compensation for deleted genes from retained syntenic paralogues in pAP146 lines. All abundance estimates are in transcripts per million. Plots A and B feature a line at Y=X indicating no change in expression in the specified comparison. A) Total expression of the retained syntenic paralogues of deleted genes in wt and pAP146 heterozygous and homozygous deletion lines. B) There is no expression of the deleted genes in the homozygous deletion lines and approximately 50% of wt expression levels in the heterozygous deletion lines. wt = wild type, which in plots A and B refers to Col-0 and sibling controls. het = heterozygous, hom = homozygous.

A

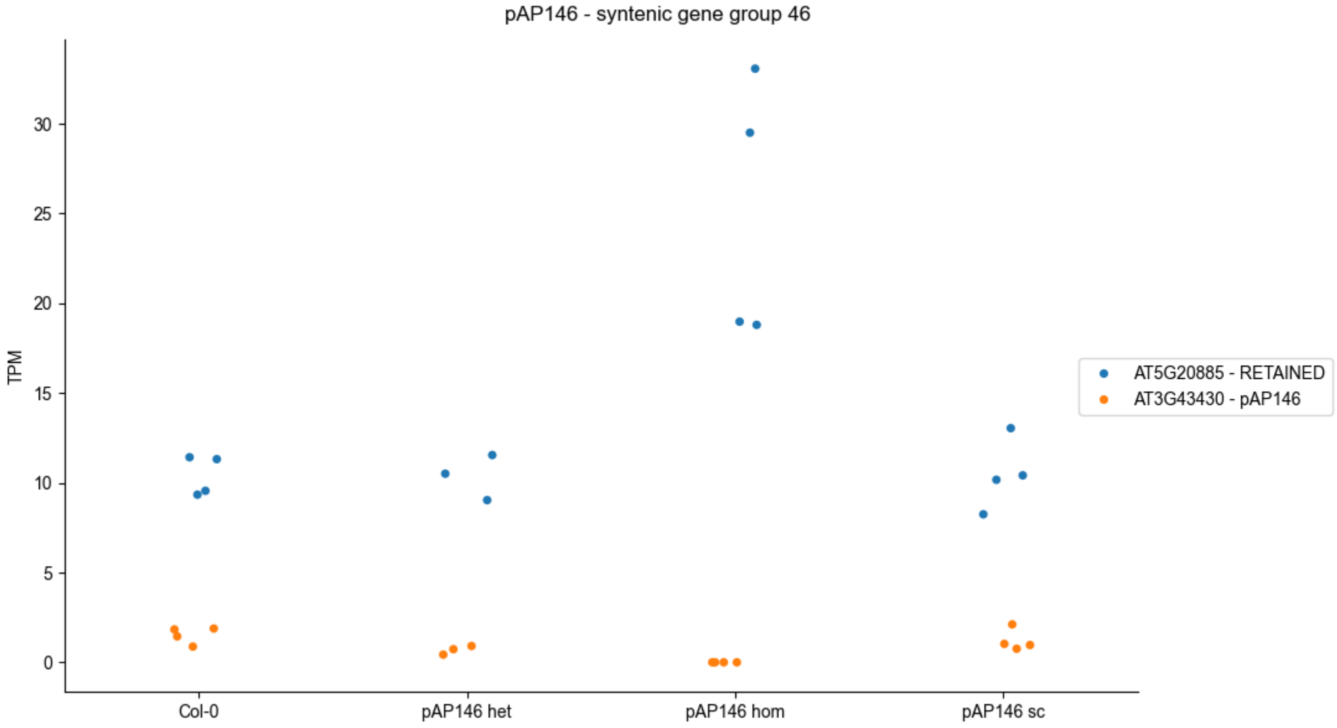

B

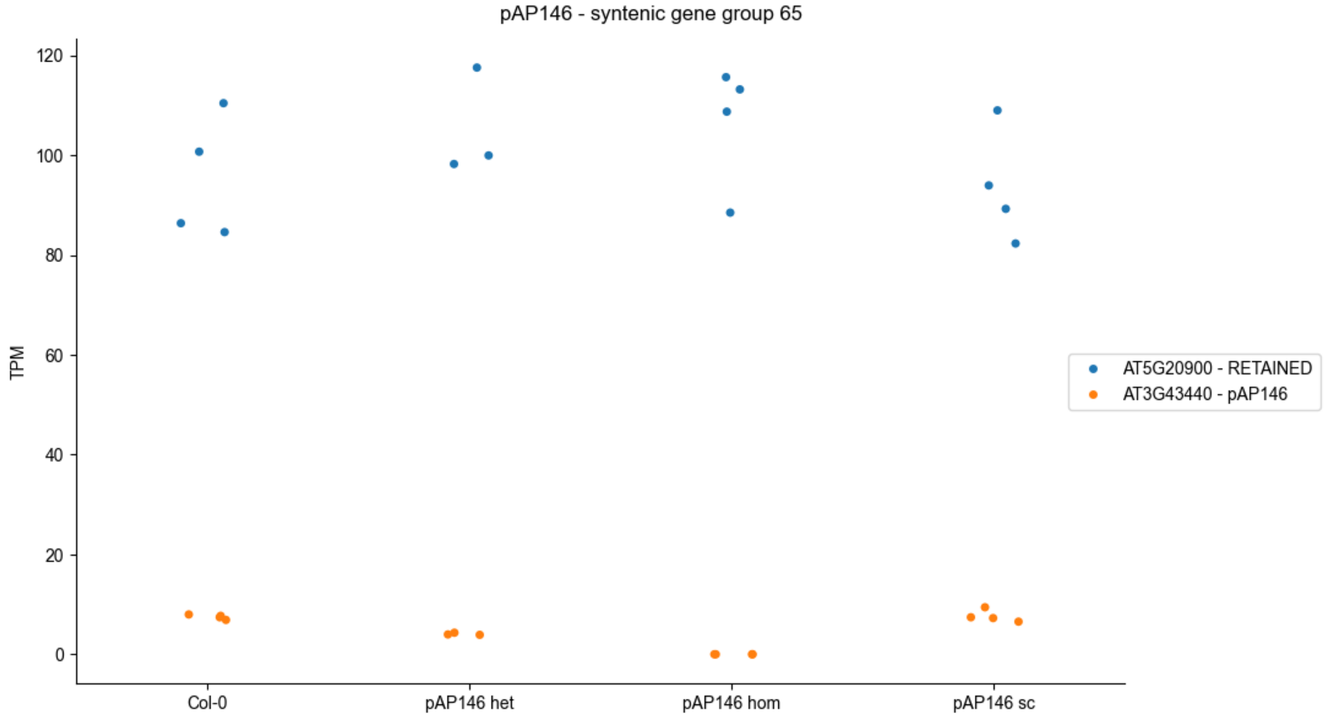

Fig. S27. A-B) Expression of syntenic paralogues in pAP146 deletion lines. Expression is indicated as TPM. Jitter has been added to the categorical "sample" axis for visualization purposes. Genes that weren't deleted are marked as "retained" in the figure. Genes that were deleted are marked with the line name (i.e., pAP146). het = heterozygous, hom = homozygous, sc = sibling wt control.

Table S2. Centrality of deleted gene lists

	Observed	p-value	logFC
Block 271	9.503178	0.134	-1.201768
Block 438	16.74896	0.6144	-0.392766
Block 268	27.677781	0.4102	0.337088
Block 324	25.421745	0.6385	0.21151

Deleted genes are not significantly more central or peripheral than expected. For each list of deleted genes, we calculated the weighted node connectivity (WNC), a measure of network centrality, and compared it to a bootstrap distribution generated by sampling random gene lists of equal length. The table includes the following columns: Observed, the WNC for that list; p-value, the proportion of bootstrap samples with a WNC showing a greater absolute deviation from the bootstrap mean; and logFC, the log fold change between the observed WNC and the bootstrap mean.

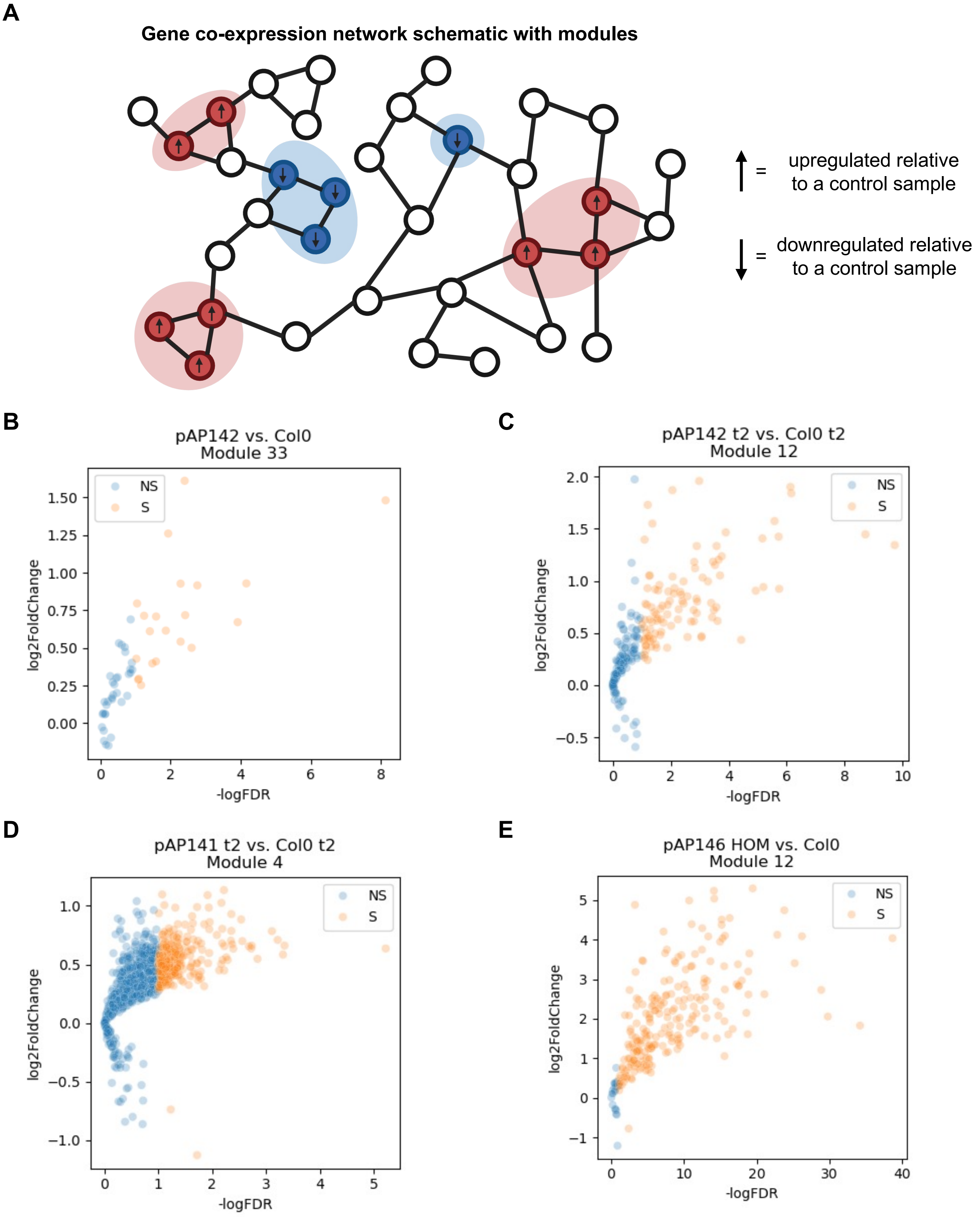

Fig. S28. A) An illustration of a sample co-expression network with annotated modules (light red or blue circles/ovals) containing certain groups of genes (circles). Genes/modules in red indicate upregulated genes relative to controls, while those in blue indicate downregulated genes. Diagram was created with BioRender.com B-E) Volcano plots depicting significance of differential expression vs. change in expression for upregulated DEGs from deletion lines that are enriched in specific network modules. In volcano plots, t2 = time point 2, hom = homozygous, NS = not significant, S = significant.

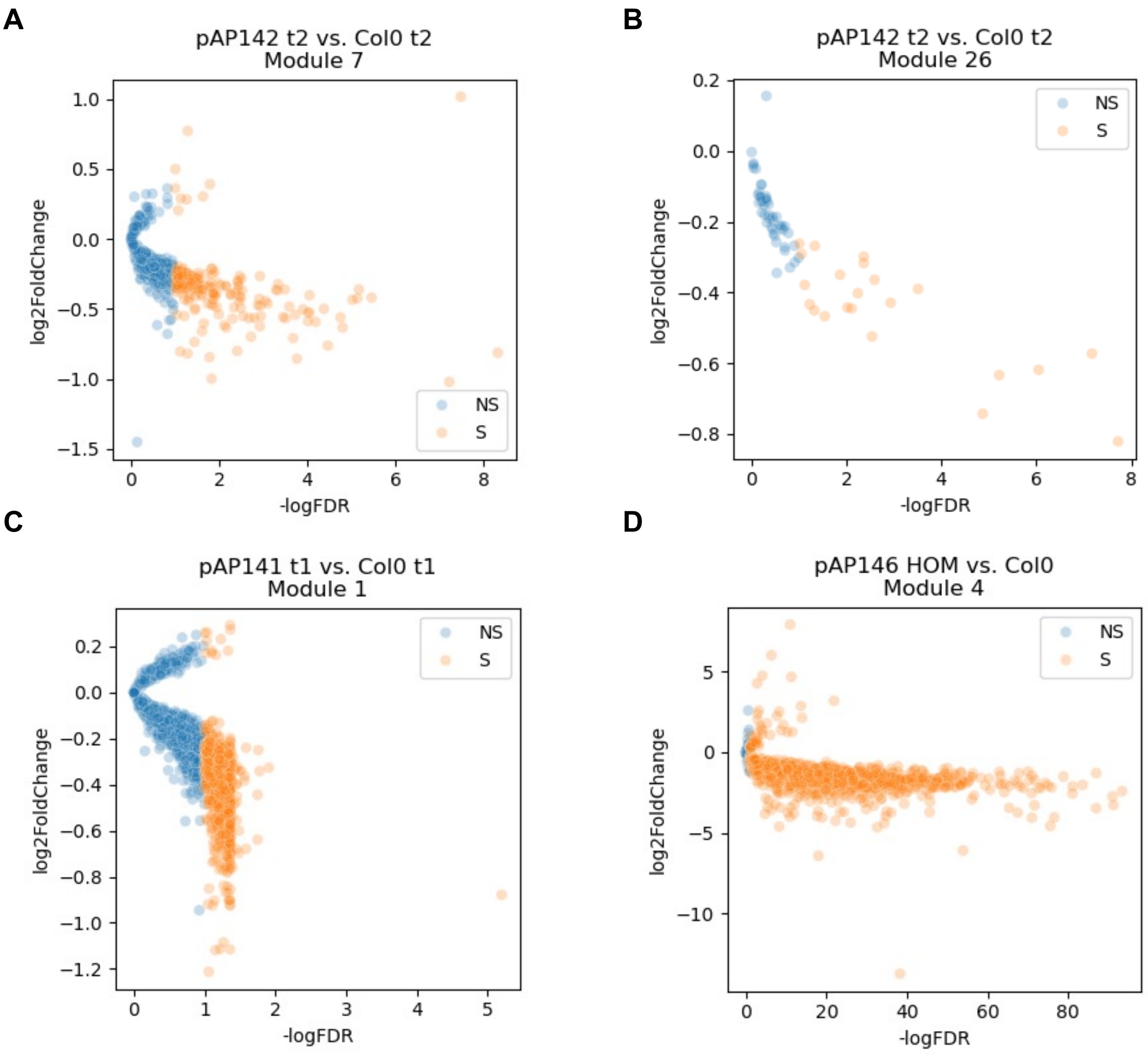

Fig. S29. A-D) Volcano plots depicting significance of differential expression vs. change in expression for downregulated DEGs from deletion lines that are enriched in specific network modules. In volcano plots, t1 – time point 1, t2 = time point 2, hom = homozygous, NS = not significant, S = significant.

Dataset S1 (separate file). Descriptions of syntenic blocks and deleted blocks.

Dataset S2 (separate file). Descriptions of genes and TEs within deleted regions and their paired blocks. Syntenic paralogues and tandem duplicates are also included. Gene expression fold change values and adjusted p-values are also provided for syntenic paralogues of genes within deleted regions.

Dataset S3 (separate file). gRNA spacer and off-target information.

Dataset S4 (separate file). Differential expression analysis table.

Dataset S5 (separate file). Total number of differentially expressed genes that are either upregulated or downregulated in each deletion line. List of deleted or differentially expressed genes and their enrichment in particular gene network modules.
